# Charting development of ERP components on face-categorization: Results from a large longitudinal sample of infants

**DOI:** 10.1016/j.dcn.2020.100840

**Published:** 2020-08-16

**Authors:** Renata Di Lorenzo, Carlijn van den Boomen, Chantal Kemner, Caroline Junge

**Affiliations:** a Experimental Psychology, Helmholtz Institute, Utrecht University, the Netherlands; b Developmental Psychology, Utrecht University, the Netherlands; c Brain Center Rudolf Magnus, University Medical Center Utrecht, the Netherlands

**Keywords:** Face categorization, ERPs, Infants, N290, Longitudinal

## Abstract

From infancy onwards, EEG is widely used to measure face-categorization, i.e. differential brain activity to faces versus non-face stimuli. Four ERP components likely signal infants’ face-sensitivity but reflect different underlying mechanisms: the P1, N290, P400, Nc. We test whether these components reveal similar developmental patterns from early to late infancy, using a longitudinal dataset of 80 infants tested at 5 and 10 months. The P1, N290, and the Nc show face-categorization already in 5-months-olds, a pattern which did not change over time. Development is visible as increased amplitudes in all components, but similar for face and non-face stimuli. By using Markov models, we illustrate that there are differences in the distribution of individual trajectories of face-categorization components from 5 to 10 months. Whereas individual trajectories appear more varied for the Nc and the P1, the N290 reveals a more consistent pattern: a larger proportion of 5-month-olds shows the dominant group response; a larger proportion of 10-month-olds remains in this group, and larger proportions of the alternative trajectories from 5- to 10-month-olds move towards the dominant group. This is vital information when one wants to examine individual differences in infant ERPs related to face-categorization.

## Highlights

• We report longitudinal ERP data of 80 infants in a face-discrimination task.
• P1, N290, Nc are all sensitive to faces in five-month-olds.
• P1, N290, Nc show equal face-categorization in infants tested longitudinally.
• N290 shows less variation in face-categorization trajectories than P1 or Nc.
• Visual ERPs increase in amplitude over infancy, but this is not face-specific.

## 1 Introduction

The ability to discriminate human faces from objects, here referred as face-categorization, is fundamental for survival. Already in infancy, research shows that 3-month-olds consistently prefer to look at human faces (cf. Libertus et al., 2017). This preference is mirrored in neural measures, such as in event-related potentials (ERPs). To date, numerous studies used ERPs as this method allows to investigate face-categorization using the same task (e.g., passively viewing stimuli) across ages. Nevertheless, ERP components obtained from infants often differ from adults, varying in timing, distribution, or even polarity. It remains difficult to track development of these ERP components in infancy, as most studies report either results from a single age-group or use a cross-sectional design (e.g., Conte et al., 2020). The current study therefore reports longitudinal data from 80 infants tested at five and at 10 months using a simple visual contrast: human faces and houses.

Four ERP-components have been linked to different aspects of face-processing in infants (Conte et al., 2020): the P1, the N290, the P400 and the Negative central (Nc; de Haan et al., 2003). The earliest component P1 is not face-specific but associated with differences in low-level visual features that exist between face and non-face stimuli (Rossion and Jacques, 2008; Conte et al., 2020). The N290 reflects the encoding of faces specifically, and is thought to be the precursor of the adult face-sensitive component N170 (de Haan et al., 2003; Conte et al., 2020). A similar differentiation between face and non-face stimuli as observable in the N290 is often also detectable in the mid-latency peak P400, but not always, making it difficult to understand what this component reflects (Conte et al., 2020). Some consider the P400 to be a corollary of the N290 (Halit et al., 2003), with the complex eventually integrating into the adult N170. Others suggest that the P400 is additionally involved in detection of unfamiliar faces (e.g., Scott and Nelson, 2006). Finally, there is the mid-latency Nc (with an opposite polarity to the P400), which is not face-specific but is often included in face-processing studies as it reflects a child’s heightened attention allocation to faces (Courchesne et al., 1981; de Haan and Nelson, 1997; Reynolds and Richards, 2005).

For each component there is evidence suggesting that it signals face-categorization. Nevertheless, as reported in Table 1, infant ERP studies contrasting face versus non-face stimuli do not always concur in their findings. For instance, some studies report amplitude differences between face and non-face stimuli at the P400 (Guy et al., 2016; Jones et al., 2016), while others do not show such an effect (Halit et al., 2004; Xie and Richards, 2016). Such inconsistencies obscure our understanding of face-categorization development. There are several possibilities why findings are contradictory. First, most studies used small sample sizes, making it difficult to reproduce findings (Frank et al., 2017). Moreover, research often reports on single age groups or cross-sectional samples, but not within-subjects, thus ignoring individual trajectories (cf. Luyster et al., 2014; Webb et al., 2005; Yrttiaho et al., 2014). Studies also differ in the type of contrast employed or in calculations of components, which questions whether we can align results. Finally, most studies report only a subset of ERP components denoting face-categorization, which compromises comparison across studies. Consequently, to chart development the field requires large longitudinal infant studies that report all four ERP components related to face-categorization.

**Table 1:** Overview of infant studies on face-categorization (i.e., explicitly contrasting ERP responses to human faces versus contrast stimuli), ordered by age of participants. Dashes denote that the peaks were not reported; n.s. indicates no significant effect; > indicates absolute higher amplitude or longer latency; F is used for human face and C for contrast stimuli. ^a^Results were only significant for the right hemisphere. ^b^Amplitude was greater for faces only when selecting attention periods based on heart-rate measures. ^c^Between-subjects design counterbalancing face- and contrast-category. ^d^Group of infants at low likelihood for ASD. ^e^N = 57 consisted of: 21 infants at low likelihood for autism spectrum disorder (ASD).

Study	Age in months	Participants included	ContrastCategory	P1		N290		P400		Nc	
				Ampl	Lat	Ampl	Lat	Ampl	Lat	Ampl	Lat
Macchi Cassia et al., 2006	3	15	Top-heavy face/configuration	n.s.	n.s.	n.s.	n.s.	n.s.	n.s.	n.s.	n.s.
Gliga and Dehaene-Lambertz, 2005	3	16	Bodies	n.s.	n.s.	F > C	n.s.	n.s.	n.s.	–	–
Halit et al., 2003	3	25	Monkey faces (upright/inverted)	–	–	F > C	F < C^a^	F > C^a^	F < C	–	–
Halit et al., 2004	3	13	Visual noise	–	–	F > C	n.s.	n.s.	F > C	–	–
Peykarjou and Hoehl, 2013	3	14	Cars (upright/inverted)	n.s.	n.s.	n.s.	n.s.	n.s.	n.s.	–	–
Guy et al., 2016	4.5, 6, 7.5	14−19 per age group	Toys (novel/familiar)	–	–	n.s.^b^	n.s.	F < C	n.s.	n.s.	–
Parise et al., 2010	3, 4	16−15	Scrambled faces	n.s.	n.s.	n.s.	n.s.	–	–	n.s.	F < C only 3 months
de Haan & Nelson, 1999	6	44^c^	Toys (novel/familiar)	n.s.	n.s.	n.s.	n.s.	n.s.	F < C	n.s.	n.s.
de Haan et al., 2002	6	34^c^	Monkey (upright/inverted)	–	–	F > C	n.s.	n.s.	n.s.	–	–
Jones et al., 2016	6, 12	50 − 59^d^ per age group	Objects	–	–	–	–	F < C	F < C	F < C	–
Peykarjou et al., 2014	9	49	Monkey	F < C	n.s.	n.s.	F > C	F > C	F > C	–	–
Xie and Richards, 2016	3, 4.5, 6	12 per age group	Toys	–	–	F > C only 6 months	–	n.s.	–	F > C	n.s.
Webb et al., 2005	4,6,8,10,12	16−28 per age group	Toys (novel/familiar)	–	–	–	–	–	–	F < C	F < C only 12 months
Conte et al., 2020	4.5, 6, 8,12	12−26 per age group	Toys	F > C	–	F > C	–	n.s.	–	F < C 4months F > C 6-8months	–
McCleery et al., 2009	10	20^d^	Toys (novel/familiar)	n.s.	n.s.	F > C	n.s.	F < C	F < C	F < C	n.s.
Guy et al., 2018	12	57^e^	Toys (novel/familiar)	–	–	F > C	n.s.	n.s.	n.s.	n.s.	–
Halit et al., 2003	12	26	Monkey faces (upright/inverted)	–	–	F > C	F > C	n.s.	F < C	–	–

Why is it essential to understand how these ERP components develop? Face-processing has been put forward as a key marker of social development (Dawson et al., 2005). Already in infancy, abnormalities in face-processing are evident in children with neurodevelopmental disorders such as autism spectrum disorder (ASD; e.g., Dawson et al., 2005; McCleery et al., 2009; Webb et al., 2011). There is also variation within typically-developing infants, the impact of which is not yet fully understood. For this we need prospective longitudinal studies such as the on-going YOUth study, which includes ERP measurements on face-categorization collected at five and 10 months (cf. van Onland-Moret et al., under review). The goal of YOUth is to study whether infant neurocognitive development, including face-categorization, can predict later social competence. Grasping development will thus allow us to separate immature from mature responses, and typical from atypical responses (see also Luyster et al., 2014).

Our study contributes to research elucidating whether there is development in face-categorization across infancy (e.g., Conte et al., 2020; Webb et al., 2005). There is reason to believe that late infancy marks a pivotal stage for face-processing. The second semester marks perceptual narrowing in face-processing to own species (Pascalis et al., 2002). This leap in development might also be mirrored in basic face-processing skills, such as face-categorization. However, results are inconclusive. As not all infant studies include a contrasting category to which the category of faces is compared to, it remains difficult to assess whether maturation in any component involved in face-processing (e.g., P1, N290, P400 or the Nc) is specific to faces (e.g., Taylor et al., 2004) or whether it holds equally across visually-evoked potentials (Conte et al., 2020; Kuefner et al., 2010).

Previous studies report face-categorization abilities from three months of age (cf. Libertus et al., 2017; Table 1). Hence, we expect ERP markers to signal face-categorization at five months, and to remain equally (or increasingly) sensitive at 10 months of age. Developmental change in face-categorization should manifest itself as interactions between stimulus type with age.

Development might also be observed with a larger proportion of older infants showing mature responses. We use descriptive Markov models to visualize the individual trajectories from early to late infancy in order to compare development across the different ERP-components. For each component we group 5-month-olds into three categories based on the mean face-house difference (negative difference, no difference, or positive difference). We then quantify their transition patterns to each of these groups at 10 months. If there is no development, most infants should remain in the same group at both visits. A component signals development when more 10-month-olds move into the dominant group that already showed a difference in face-categorization at five months. Another possibility would be the case with multiple trajectories without any dominant pattern. In this case, this component does not solely reflect face-categorization. We predict that there are more consistent patterns in the developmental trajectories concerning the N290/P400 (related to face perceptual processing) than in the Nc (related to attention); for the P1 we had no clear hypothesis.

To summarize, we use a large longitudinal dataset to advance our understanding on infant face-categorization by testing whether face-categorization components change between five and 10 months. As the examined components are related to distinct stages of face-processing, comparing their developmental trajectories further yields insights whether the underlying mechanisms develop similarly. This is vital information when one wants to start interpreting individual differences in face-categorization.

## 2 Material and methods

### 2.1 Participants

As part of the YOUth cohort (cf. van Onland-Moret et al., under review), we tested 173 healthy infants around five months of age who returned to our center when they were around 10 months old. We excluded children because they were born pre-term (<36 weeks; *n* = 5); because after pre-processing their EEG contained less than 10 trials per condition to calculate one of the ERP components (*n* = 81; see preprocessing steps below); or due to technical problems (*n* = 2). There were 75 infants who contributed data to all four components at both visits (39 girls), and an additional 10 infants who contributed data either to the P1, N290 and P400 components (*n* = 5; 3 girls) or to the Nc component (*n* = 5; 2 girls). As a result, all four components comprised data from 80 infants (see Table 2 for information on the participants’ age at each visit). The study was approved by the medical ethical committee of the University Medical Center Utrecht, in accordance with the Declaration of Helsinki.

**Table 2:** Mean age at visit 1 and 2, and mean age difference between the two visits of infants providing EEG data for the N290/P400 and for the Nc. Age is reported in days, standard deviations are shown in the brackets.

Component	Age visit 1	Age visit 2	Age difference between visits
P1/N290/P400	168 (22.5)	319 (25.7)	151 (34.2)
Nc	168 (22.0)	318 (25.7)	150 (33.5)

### 2.2 Stimuli

**Fig. 1:**
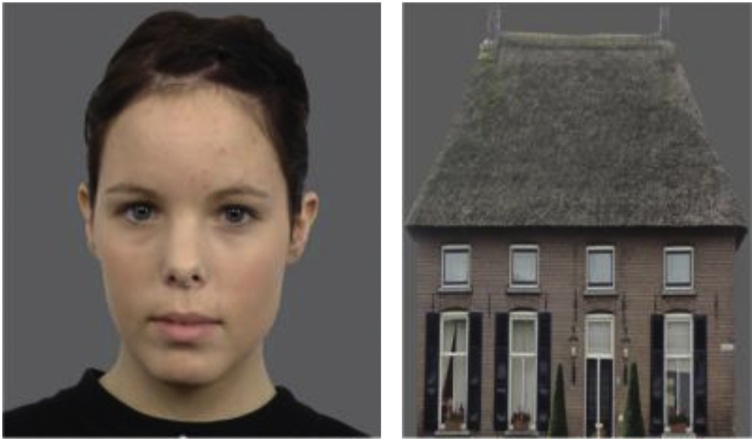
Example of face and house stimuli displayed in the task.

Stimuli were coloured pictures of six female and six male models with a neutral expression selected from the Radboud Faces Database (females identities: 12, 22, 26, 27, 37, 61; males identities: 7, 15, 25, 36, 49, 71; Langner et al., 2010) and 12 coloured pictures of typical Dutch houses selected from the internet (for an example see Fig. 1; the full set of stimuli is reported in the supplementary materials). The stimuli were depicted on a grey background (RGB: 108) and measured 20.5 cm width × 22.5 cm height (visual angle: 19.4°× 21.2°). During the inter-stimulus intervals (ISI) infants saw a 5.3 × 5.3 cm square in the middle of the screen, which was composed of four coloured squares (red, yellow, blue and green; visual angle: 4.7° × 4.7°).

### 2.3 Procedure

During the study infants sat on their parent’s lap or on a high chair at approximately 65 cm distance from a 23-inch computer monitor (refresh rate 60 Hz, 1920 × 1080 resolution). Below the screen there was a webcam camera sampling at 15 Hz to record the child’s looking behaviour during the testing session. The testing room was semi-dark, controlled for luminance (between 8−20 lux) and temperature (between 18−25°).

During the task infants passively watched trials consisting of pictures of (neutrally-looking) faces or houses. The task was programmed in Matlab using Psych-Toolbox 3 (Brainard and Vision, 1997). Trial duration was 1000 ms followed by a jittered ISI between 700 and 1000 ms. In total, there were 96 trials: 48 face trials (4 × 12 models) and 48 house trials (4 × 12 houses). The stimuli order was pseudo-randomized: per block of 24 trials (4 blocks in total) all pictures appeared once in a randomized order. The experimenter played additional sounds or video clips to redirect the child’s attention to screen. The experiment ended when all the 96 trials were presented or until the baby was too distracted or fussy to attend. The task lasted approximately 3−4 min. Parents were instructed not to interact with their child during the experiment.

### 2.4 ERP recording

Continuous EEG was recorded at a 2048 Hz sample rate using a 32-channel ActiveTwo BioSemi system (Actiview version 7.05; Amsterdam, the Netherlands). Electrodes were positioned at standard EEG recording locations according to the international 10–20 system (28 lateral channels: FP1/2; F7/8; F3/4; AF3/4; FC1/2, FC5/6, C3/4, T7/8, CP1/2, CP5/6, P3/4, P7/8, O1/2, PO3/4; 4 midline channels: Fz, Cz, Pz, Oz). Two electrodes, the Common Mode Sense and the Driven Right Leg, provided the active ground. Electrode offsets were less than 20 nV.

### 2.5 Data analyses

#### 2.5.1 Preprocessing

EEG data were pre-processed using Brain Vision Analyzer software (version 2.1; Brainproducts, GmbH). Data were first down-sampled offline to 512 Hz, and filtered with a high-pass filter of 0.1 Hz (24 dB/oct), a low-pass filter of 30 Hz (24 dB/oct) and a notch filter of 50 Hz. Continuous EEG data were reduced to epochs of 200 ms pre-stimulus until 1000 ms post-stimulus, with a baseline correction of −150 ms to 0 s. We removed whole trials manually when the child looked away from the screen between 0 and 600 ms after stimulus onset. Subsequently, we removed trials from single electrodes when an artifact was found between 0−600 ms post-stimulus. Artifacts were defined as amplitudes +/ −200 μV; as a difference of less than 3 μV within a moving window of 200 ms; or as a voltage change of more than 50 μV per sampling point. An electrode was rejected if there were less than 5 artifact-free trials (this criterion also holds to electrodes of interest). We removed whole trials when more than 16 % of electrodes contained artifacts (based on previous research on face processing in infants, e.g., Halit et al., 2003; van den Boomen et al., 2017). Finally, we referenced the activity of each single active electrode to the average of all included electrodes before calculating the event-related potential per condition per electrode.

Participants were included in the statistical analyses if the final average per experimental condition contained at least 10 trials for critical electrodes (i.e., for the P1: PO3, O1, Oz, O2, PO4; for the N290/P400: P3, PO3, O1, Oz, O2, PO4, P4; for the Nc: Fz, C3, C4^1^ ; cf. Kuefner et al., 2010; Munsters et al., 2017; van den Boomen et al., 2017). The average number of included segments was 30 per condition (P1/N290/P400: mean 31.6 trials (range 13–47) and 28.8 trials (range 11–47) at first and second visit, respectively; Nc: mean 31.7 trials (range 13–47) and 28.6 trials (range 11–47) at first and second visit, respectively).

#### 2.5.2 Component analyses

Because we are interested in face-categorization, that is, the difference between processing faces relative to houses, we required both conditions in our analyses (Luck and Gaspelin, 2017). For each component (P1, N290, P400, Nc) we chose to report first as our main dependent variable mean amplitude rather than latency of each component because it can be difficult to calculate peak latency for mid-latency components as infant ERPs are characterized by greater slow wave activity resulting in broad peaks in their ERPs (DeBoer et al., 2007).

For each ERP component, we selected time windows of interest based on previous research on infant face processing as well as by checking individual averaged waveforms. For the P1, this was 90−180 ms (Macchi Cassia et al., 2006; Luyster et al., 2014), for the N290 170–300 ms (de Haan and Nelson, 1999), for the P400 300–500 ms (de Haan and Nelson, 1999), and for the Nc 300–600 ms (Munsters et al., 2017). We averaged the mean amplitude over all critical electrodes per condition per recording session: for the P1, this was averaged over five posterior electrodes (PO3, O1, Oz, O2, PO4); for the N290/P400 over seven occipito-parietal electrodes (P3, PO3, O1, Oz, O2, PO4, P4); and for the Nc over three fronto-central electrodes (Fz, C3, C4).

In addition, we computed the amplitude difference between the mean amplitude of the P1, N290 and P400 components component of interest and the peak of its preceding component to test if face-house differences at these components partly reflect carryover effects from preceding peaks (so-called ‘peak-to-trough- analyses’; for similar tests see Conte et al., 2020). Specifically, we calculated peak-to-trough differences between the P1 and the preceding negative peak (i.e. N80 peak; mean amplitude was extracted between 70−90 ms post stimulus onset at PO3, O1, Oz, O2, PO4); between the N290 and the preceding P1; and between the P400 and the preceding N290, using the ERP averages obtained from each participant at each visit, electrode of interest and condition.

Besides the analyses reporting mean amplitude within a fixed time window for each component, we also add latency analyses for those earlier components for which peak latencies could be determined: for the P1, latencies for both face and house conditions, and for the N290 only for the face condition, as there were no clear peaks detectable for the house conditions. Peak latency of the P1 (for houses and faces) and N290 (for faces) were calculated as the moment in time when a maximum positive or negative peak occurred at critical electrodes within the time windows of interest. We then averaged the peak latency across electrodes of interest, separately for each visit and participant.

#### 2.5.3 Statistical analyses

For each component we first carried out repeated measures ANOVAs, with mean activity on critical electrodes as our dependent variable, and Stimulus type (faces versus houses) and Visit (Visit1; Visit2) as within-subject factors. We then conduct peak-to-trough analyses to see whether observed effects arise from carryover effects from preceding peaks.

Note that our Supplementary materials contain additional analyses on mean amplitudes. First, we repeat analyses that include Electrode as a factor (i.e., dependent variables are now mean amplitudes per electrode per stimulus type per visit). Second, analyses are repeated with possible co-variates, as our large sample of infants shows potential variation in certain other subject characteristics relevant to face-categorization, which are not of interest to our main research question, but might be worthy of interest to others (i.e., age at visits; time interval between the two visits; and the number of included trials as a proxy for the signal: noise ratio). The supplementary materials also contain the Brown-Forsythe tests indicating that for each component there is similar variation (and therefore noise) between the two visits in the face-house difference amplitude.

Our second set of analyses visualizes individual trajectories of development, using Markov models. We formed an index of face-sensitivity for each component for each infant at each age by subtracting the mean amplitude for houses from that of faces. Next, based on the amount and direction of this difference score, we divided the 5-month-olds into three subgroups: no difference between faces and houses (between − /+ 1.5 μV); a positive difference (i.e., faces versus houses elicited a larger than 1.5 μV positive response); or a negative difference (i.e., faces versus houses elicited a larger than 1.5 μV negative response). The second author - blind to the results of the current study - defined the thresholds by reviewing the observed differences in previous research on face-sensitivity (Halit et al., 2003; Munsters et al., 2017; van den Boomen et al., 2017; Webb et al., 2005). Note that these thresholds are arbitrary and open to discussion, and thus should not be considered absolute but rather be used as an indication of meaningful differences. Once we categorized the infants’ responses at the first visit into separate subgroups, we calculated for each component the transitional probabilities of a child either remaining in the same group or moving to one of the other two groups at the second visit (using the same thresholds).

Finally, we report latency tests on the P1 (face, house) and the N290 (face only). For the P1, we performed a repeated measures ANOVA with peak latency as dependent variable and Stimulus Type and Visit as independent variables. To test development in the latency of the N290, we conducted a paired *t*-test on the peak latency averaged across channels for each visit (i.e., latency to faces at visit1 vs visit2). The Supplementary materials repeat these tests on single electrodes of interest.

## 3 Results

**Fig. 2:**
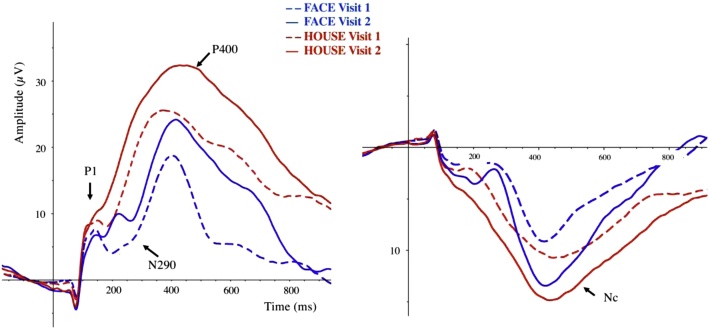
Grand-averaged waveforms obtained at Visit1 (dashed lines) and Visit2 (solid lines) in response to faces (blue lines) and houses (red lines). The plot represents the mean activity recorded, for the left panel, from parieto-occipital electrodes (P3, PO3, O1, O2, Oz, PO4, P4); while for the right panel from fronto-central electrodes (Fz, C3, C4).

For each component, we first report the results on the mean amplitudes for faces versus houses across infancy from the ANOVAs, followed by the accompanying Markov model that visualizes the individual trajectories. In addition, for the early components P1 and N290 we end with the latency tests. Fig. 2 depicts the ERPs time-locked to the onset of faces and houses for the first and second visit, for the P1, N290 and the P400 on occipito-parietal electrodes (left), and for the Nc on fronto-central electrodes (right). (Figures plotting all electrodes for those 75 infants who contributed enough data for all analyses are included in the Supplementary materials, separated by visit). Table 3 synthesizes results from the Markov models for all four components of interest: It reports for each of the created subgroups the sample sizes, followed by the average difference in amplitude (face-house) plus range, for visit 1 and 2, respectively.

**Table 3:** Descriptive subgroup summaries for each of the three components denoting face-categorization (face – house amplitude), split by age. Note: All values are in μV and are based on the difference in mean amplitude of the ERP component for face minus houses; n = subgroup size, Min. = minimum, Max. = maximum, SD = standard deviation.

	Visit1					Visit2				
P1	n	Mean	Min.	Max.	SD	n	Mean	Min.	Max.	SD
Negative difference	44	−6.83	−16.7	−1.63	3.92	53	−7.82	−18.13	−1.64	4.86
No difference	14	−0.34	−1.48	1.26	0.98	13	−0.34	−1.26	0.77	0.70
Positive difference	22	6.95	1.76	19.57	4.99	14	8.05	2.27	22.6	6.59
**N290**										
Negative difference	66	−10.94	−27.06	−1.73	6.22	71	−12.00	−35.08	−2.15	7.53
No difference	5	0.19	−0.45	1.01	0.63	4	−0.07	−0.57	1.24	0.87
Positive difference	9	5.80	2.46	10.54	2.53	5	7.97	2.20	17.88	5.89
**P400**										
Negative difference	68	−11.87	−26.85	−1.60	6.59	66	−12.97	−31.12	−2.73	6.46
No difference	5	−0.40	−1.39	1.13	1.05	7	0.22	−1.41	1.39	1.12
Positive difference	7	7.10	3.66	10.90	3.08	7	5.83	1.58	12.34	4.13
**Nc**										
Negative difference	14	−4.16	−14.27	−1.51	3.32	14	−6.93	−25.91	−2.03	6.12
No difference	18	0.16	−1.40	1.36	0.93	17	0.09	−1.36	1.38	0.91
Positive difference	48	6.17	1.51	19.63	4.42	49	6.19	1.55	13.62	2.92

### 3.1 P1 amplitude

The repeated measures ANOVA indicates a main effect of Stimulus type (*F*(1,79) = 30.2, *p* < .001, *η_p_^2^* = .28), due to houses (*M* = 10.5 μV, *SD* = 6.76) eliciting larger amplitudes than faces (*M* = 7.65 μV, *SD* = 6.14). No other effect reached significance (all *p*s>.14).

Peak-to-trough analysis confirmed the main effect of Stimulus type (*F*(1,79) = 68.0, *p* < .001, *η_p_^2^* = .46; houses: *M* = 14.03 μV, *SD* = 5.44; faces: *M* = 11.21 μV, *SD* = 4.75). Furthermore this analysis shows a main effect of Visit (*F*(1,79) = 6.65, *p* = .012, *η_p_^2^* = .078): in Visit2 P1-amplitudes (*M* = 13.61 μV, *SD* = 6.25) were larger than in Visit1 (*M* = 11.64 μV, *SD* = 5.63).

**Fig. 3:**
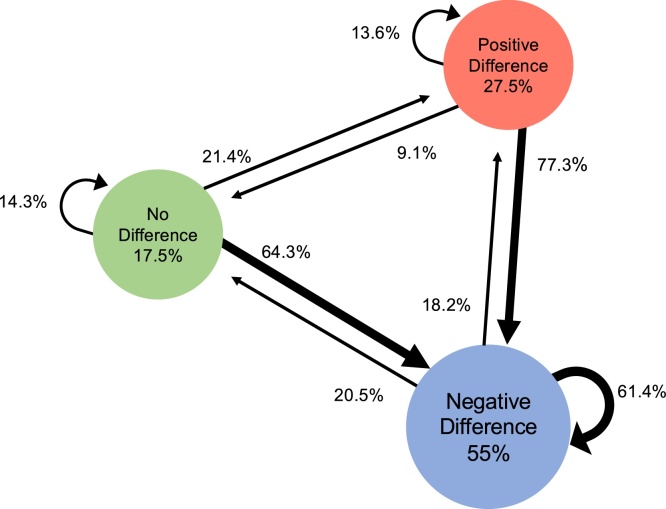
Markov model depicting the transition trajectories observed across the two Visits for the P1 amplitude denoting face-categorization. Circles report the percentage of infants that at Visit1 show either no difference between face-house amplitudes (green), a negative difference (blue), or a positive difference (red). The circular arrows indicate the percentage of infants that at Visit2 remain in a category, while straight arrows indicate the percentage of infants that moved from one category at Visit1 to another at Visit2.

Fig. 3 plots the Markov Model for P1, which highlights that all trajectories are possible. In line with the results from the first ANOVA, the dominant group pattern is a negative face-house difference (i.e. faces elicit a less positive P1 than houses): there are 27 children out of 80 (33.75 %) who show this pattern at both visits. The remaining children show nearly all possible trajectories, yet most of the infants not showing the dominant negative pattern at Visit1 display the negative difference at Visit2 (*n* = 26; 72 %).

### 3.2 P1 latency

Table 4 reports mean and standard deviation of the P1 latency elicited by faces and houses at the two visits. The ANOVA shows no significant effect of Stimulus type, Visit nor interaction (all *p*s>.262). Thus, it appears that the latency of the P1 does not change over time nor is affected by stimulus type.

**Table 4:** Mean and SD of P1 peak latency elicited by faces and houses at first and second visit.

	Visit 1		Visit 2	
	Mean (ms)	SD	Mean (ms)	SD
Face	135	15.9	132	18.6
House	134	17.9	132	18.6

### 3.3 N290 amplitude

For the N290, the repeated measures ANOVA shows that there is a main effect of Stimulus type (*F*(1,79) = 209, *p* < .0001, *η_p_^2^* = .73): for both visits, faces elicited a less positive N290 (i.e. more negative; *M* = 7.31 μV, *SD* = 6.91) than houses (*M* = 16.6 μV, *SD* = 6.81). There is also a main effect of Visit (*F*(1,79) = 16.0, *p* < .0001, *η_p_^2^* = .17), with infants at Visit1 showing a smaller positive mean amplitude across both stimulus types (*M* = 9.60 μV, *SD* = 7.28) than infants at Visit2 (*M* = 14.3 μV, *SD* = 8.90). There is no interaction between Visit and Stimulus type (*F*(1,79) = 1.60, *p* = .21, *η_p_^2^* = .020).

Peak-to-trough analysis confirms that both main effects are not carry-over effects from the P1: there is still the main effect of Stimulus type (*F*(1,79) = 98.3, *p* < .001, *η_p_^2^* = .55; houses: *M* = 8.59, *SD* = 5.82; faces: M = 1.46, *SD* = 6.47); coupled with the main effect of Visit (*F*(1,79) = 98.3, *p* < .001, *η_p_^2^* = .55; Visit1: *M* = 2.84, *SD* = 5.27; Visit2: *M* = 7.20, *SD* = 7.61). Again, no interaction emerged (*p* > .30).

**Fig. 4:**
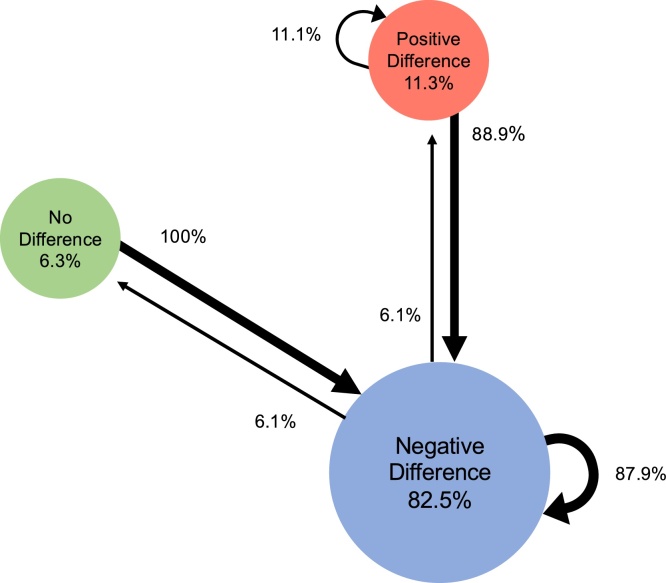
Markov model depicting the transition trajectories observed across the two Visits for the N290 amplitude denoting face-categorization. Circles report the percentage of infants that at Visit1 show either no difference between face-house amplitudes (green), a negative difference (blue), or a positive difference (red). The circular arrows indicate the percentage of infants that at Visit2 remain in a category, while straight arrows indicate the percentage of infants that moved from one category at Visit1 to another at Visit2.

Fig. 4 plots the Markov model for the N290. The majority of our infants (*n* = 58; 72.5 % of the sample) shows a negative face-house difference at both visits. For those 14 infants who show either a positive or no-difference effect at Visit1, all but one has moved to the negative-difference group at Visit2. Strikingly, not all trajectories are present out of the possible trajectories; for instance, there is not one child moving from a positive face-house difference at Visit1 to a neutral difference at Visit2, which is what one would expect to occur if development is gradual, that is, from a positive difference shifting towards a negative difference.

### 3.4 N290 latency

**Fig. 5:**
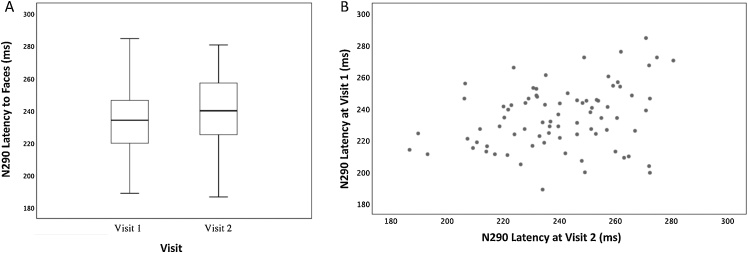
(A) boxplots of N290 latency (in ms) recorded in response to faces at first and second visit, horizontal line represents Median while the two whiskers indicate the first and third quartile; (B) scatterplot between N290 latency to faces at first and second visit.

The N290 latency was calculated within the time window 170–300 ms for each visit. The paired sample *t*-test computed to test differences in N290 peak latency averaged across electrodes in response to faces over time revealed a shorter latency at Visit1 (*M* = 234; *SD* = 19.9) compared to that at Visit2 (*M* = 240; *SD* = 21.3; *t*(79)= −2.05, *p* = .044, *d*= −.23). Fig. 5(A) shows the boxplots of N290 latency recorded in response to faces at the first and second visit; and (B) depicts the scatterplot between N290 latency to faces at the first and second visit. There are 50 infants who show a shorter latency at five months than at 10 months, whereas there are 30 infants who show the opposite pattern.

### 3.5 P400 amplitude

For the P400 amplitude (see Fig. 2 left panel), the repeated measures ANOVA shows that there is again a main effect of Stimulus type (*F*(1,79) = 207, *p* < .0001, *η_p_^2^* = .72, with houses eliciting a more positive amplitude (*M* = 26.8 μV, *SD* = 8.13) than faces (*M* = 17.0 μV, *SD* = 8.98). There is also a main effect of Visit (*F*(1,79) = 31.7, *p* < .0001, *η_p_^2^* = .29): the P400 mean amplitude increased significantly from Visit1 (*M* = 17.9 μV, *SD* = 9.53) to Visit2 (*M* = 25.9 μV, *SD* = 10.9). There is no interaction between Visit and Stimulus type (*F*(1,79) = 0.26, *p* = .61, *η_p_^2^* = .003).

Peak-to-trough analysis was conducted to ensure that the effects found at P400 were not due to carryover effects at the earlier N290. This test confirmed only the main effect of Visit, *F*(1,79) = 12.37, *p* = .001, *η_p_^2^* = .13, which was due to Visit2 (*M* = 11.68 μV, *SD* = 5.98) reporting larger amplitudes than Visit1 (*M* = 8.29 μV, *SD* = 7.41). The effect of Stimulus type is no longer significant (*F*(1,79) = 1.55, *p* = .22, *η_p_^2^* = .019).

**Fig. 6:**
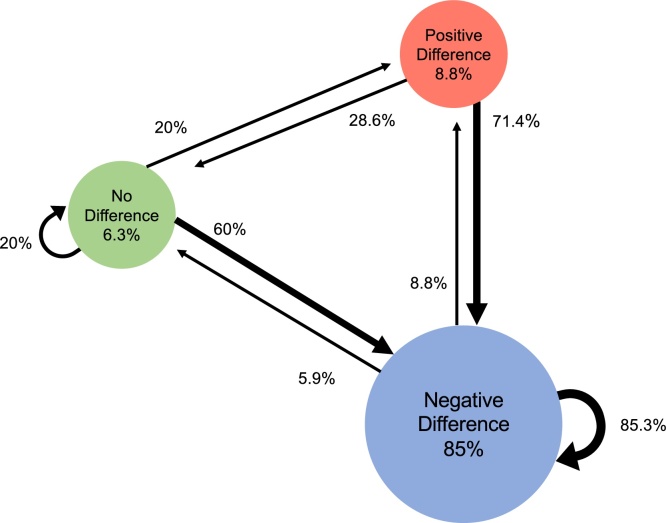
Markov model depicting the transition trajectories observed across the two Visits for the P400 amplitude denoting face-categorization. Circles report the percentage of infants that at Visit1 show either no difference between face-house amplitudes (green), a negative difference (blue), or a positive difference (red). The circular arrows indicate the percentage of infants that at Visit2 remain in a category, while straight arrows indicate the percentage of infants that moved from one category at Visit1 to another at Visit2.

Fig. 6 plots the Markov model for the P400 amplitude. Results show that the dominant group pattern is a negative difference (that is, faces elicit a less positive P400 than houses): there are 58 children (72.5 %) who show this pattern both at five and 10 months. The remaining children show nearly all possible patterns, although again, out of the 12 infants not showing a negative difference at five months, the majority (n = 8; 67 %) returns to the group dominant response at 10 months.

### 3.6 Nc amplitude

For the Nc (see Fig. 2 right panel), the repeated measures ANOVA shows that there is again a main effect of Stimulus type (*F*(1,79) = 33.6, *p* < .0001, *η_p_^2^* = .30): For both ages, faces elicited a smaller Nc (*M* = −8.50 μV, *SD* = 4.60) than houses (*M* = −11.3 μV, *SD* = 4.31). There is also a main effect of Age (*F*(1,79) = 22.4, *p* < .0001, *η_p_^2^* = .22), with infants at Visit1 showing a smaller Nc across both Stimulus types (*M* = −8.05 μV, *SD* = 4.45) than Visit2 (*M* = −11.7 μV, *SD* = 5.91). There is no interaction between Visit and Stimulus type (*F*(1,79) = 0.23, *p* = .63, *η_p_^2^* = .003).

**Fig. 7:**
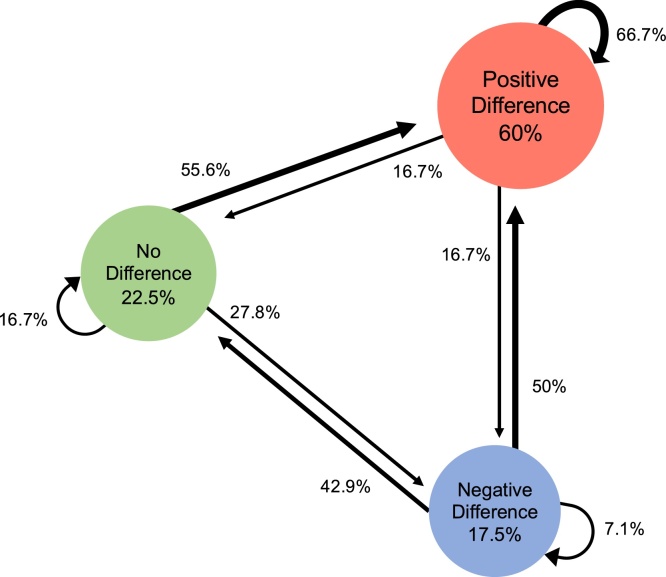
Markov model depicting the transition trajectories observed across the two visits for the Nc amplitude denoting face-categorization. Circles report the percentage of infants that at Visit1 show either no difference between face-house amplitudes (green), a negative difference (blue), or a positive difference (red). The circular arrows indicate the percentage of infants that at Visit2 remain in a category, while straight arrows indicate the percentage of infants that moved from one category at Visit1 to another at Visit2.

Fig. 7 illustrates the Markov model for the Nc. For this component, the dominant group response at both visits is a positive difference (i.e., corresponding to a smaller Nc for faces than for houses). However, compared to the two previous components, fewer children show the dominant group response at both measurements (e.g., *n* = 32, that is, 40 % of total sample). Moreover, all possible trajectories appear now to be possible, with higher rates of the non-dominant trajectories. For instance, 16 out of the 48 children who showed the dominant pattern at Visit1 (33.3 %) no longer shows this pattern at Visit2. Out of the 32 infants who did not demonstrate the dominant group response at Visit1, only 17 infants (53 %) regress to the dominant group response at Visit2.

## 4 Discussion

In the current study we analyzed longitudinal ERP data of 80 infants who came to our lab twice within a five-month-interval to participate in a face-discrimination task. Our aim was to test whether there is development of all infant ERP components previously linked to face-categorization, and to visualize this by depicting the range in trajectories from early to late infancy. Group-level results suggest that at five as well as at 10 months, face-categorization is mirrored in all infant components: the P1, the N290, the P400 and the Nc (although the effect for the P400 disappears in the peak-to-trough analyses). Crucially, the lack of interactions between stimulus-type and visit suggests no development in the processing of faces relative to non-face stimuli across infancy. Instead, the main effects of visit observed in the peak-to-trough analyses for the P1, N290, P400 and Nc indicate similar development for cortical responses to faces and non-face stimuli.

While the group-level analyses (i.e. repeated measures ANOVA on mean amplitudes) suggest that all components reflect face-categorization equally across visits, the individual-level tests (i.e. Markov models) indicate differences in the patterns of individual trajectories between the components. Specifically, for the N290 there appears to be less variation in developmental trajectories of face-categorization than for the P1 and Nc components. Below we discuss our findings in more detail, as well as mention limitations.

### 4.1 Development of components indexing face-categorization

The earliest component marking face-categorization is the P1, a component that is visible in adults (Rossion et al., 2000), children (Kuefner et al., 2010) and infants (e.g., Luyster et al., 2014). However, since the P1 is elicited by any kind of visual stimuli, this component is considered not face-specific but associated with differences in low-level visual properties that exist between face and non-face stimuli (Rossion and Jacques, 2008; Conte et al., 2020). Our types of stimuli differ in multiple ways (see supplementary materials for all stimuli). For example, while faces are presented always in frontal-view, the orientation for houses is more varied. It is therefore likely that amplitude differences in our P1 at least partly reflect sensitivity to low-level differences between faces and houses rather than face-categorization (e.g., luminance contrast, orientation, color, spatial frequency content). For instance, the P1 amplitude differs in infant responses to lower versus higher spatial frequencies (e.g., Norcia and Tyler, 1985), a property that differs between faces and houses (Jeantet et al., 2018). Note that we opted not to control for such low-level differences but for keeping the stimuli to appear as realistic as possible.

Our results further highlight that amplitude of the P1 marks equal development for both types of stimuli (in peak-to-trough analyses). This suggests that a five-month-interval is sufficient enough to warrant amplitude changes for a component indexing low-level sensory processing. This is in line with research documenting that P1 amplitude increases across infancy with age (Luyster et al., 2014). Nevertheless, we did not see this development mirrored in the latency analyses. In adults, the P1 peaks around 90–150 ms after a visual stimulus at occipital electrodes. In infants the component appears slightly delayed (e.g., our study reports 133 ms; Conte et al., 2020 124 ms), while its latency decreases slowly over childhood (Kuefner et al., 2010). Here, it seems a five-month-interval is not sufficiently large enough to detect latency changes.

The second infant component marking face-categorization is the N290, which is widely considered to be a precursor of the adult N170 (de Haan et al., 2003; Conte et al., 2020). The N170 reflects perceptual processing of faces specifically, as it has a shorter latency and larger amplitude for face stimuli (e.g., Rossion et al., 2000; cf. Dering et al., 2009). In our study, the N290 amplitude already differentiated between faces and houses at five months, a difference which remained constant at the second visit five months later. These results mirror and extend results on the N170 from Kuefner et al. (2010), who report equivalent face-categorization from 4- to 17-years. However, it is likely that face-categorization development in the N290 is more pronounced in its latency than in mean amplitude, as it is in the latency that the infant N290 differs most noticeably from the adult N170. We therefore examined the development in N290 latency to faces from early to late infancy. Surprisingly, statistical analyses suggest that the N290 peaks earlier in five-month-olds than in 10-month-olds. Nevertheless, it seems premature to rely on this outcome to conclude that N290 peaks earlier in early than in late infancy, as the difference is very small (only 6 ms). Moreover, there is quite some variation in N290 latencies across visits: while 50/80 infants show earlier N290 latencies at five months than at 10 months, a considerable number of infants (30/80) shows the opposite pattern. We therefore conclude that the N290 to faces peaks around 230−240 ms, showing little acceleration in the five-month-interval. Note that we could only assess latency scores elicited by face stimuli, but not by houses, which questions the extent to which these latency differences are specific to faces, or generalize to visual processing of multiple categories.

In infancy, the negative-going component N290 is usually followed by a positive-going peak the P400, both present at occipito-parietal electrodes. The complex N290/P400 is considered to eventually merge into the adult N170 (de Haan et al., 2003), even though the adult N170 shares polarity only with the N290. Some consider the P400 as a counterpart of the N290, whereas others consider the P400 as partly distinct from the N290 (Guy et al., 2018; for a review see Conte et al., 2020; Luyster et al., 2014). Our results reveal that with additional peak-to-trough analyses there is no evidence of additional categorization starting in the P400 time-window, making it likely that the P400 reflects residual effects of the N290. The only effect that remains is of visit: again, amplitude increases over time. More longitudinal research with older children is required to advance our understanding of how the N290 merges with the P400 into the N170 (see Picton and Taylor, 2007 for a review on cross-sectional design). For now, we conclude that our results further underscore that the N290 is a precursor to the adult N170, given its similarity in polarity and distribution.

Finally, we observed face-categorization responses at the Nc, which is a component related to attention and familiarity. It can be difficult to interpret the direction of effect at the Nc, as this component might be sensitive both to familiarity and increased allocation to one of the two contrasting categories (Reynolds and Richards, 2005). As such, the decrease of the Nc for faces versus houses reported in the current study suggests a relative familiarity with faces and/or could indicate increased attention to houses over faces. These findings replicate some (e.g., Jones et al., 2016; McCleery et al., 2009), but not all previous studies that contrasted faces versus objects (e.g., Guy et al., 2018; Xie and Richards, 2016). One of the optional factors explaining the discrepant findings between studies could be the stimuli to which faces are contrasted (see Table 1): whereas most studies presented toys (e.g. de Haan and Nelson, 1999; Guy et al., 2016, 2018; Xie and Richards, 2016), others used cars (Peykarjou and Hoehl, 2013), monkey faces (Halit et al., 2003, 2004; Peykarjou et al., 2014), both toys and houses (Conte and Richards, 2019), or just houses (current study). A differential Nc-response for face versus non-face stimuli could therefore reflect familiarity differences for some contrasting categories (e.g., human faces over monkey faces), while for other categories it might reflect differences in attention (e.g., toys versus faces; Webb et al., 2005; Xie and Richards, 2016). The variation in developmental trajectories in the Markov model for the Nc further illustrate the discrepancy in findings, which suggests that a variety of factors (e.g., stimulus type, variation within stimulus category, attention, and familiarity) affect the Nc amplitude (Conte et al., 2020). Indeed, one possibility is that while the mechanism(s) underlying Nc (e.g., attention or preference allocation) are stable throughout infancy, it is the attention towards the different stimuli types that changes with age or over the course of an experiment (Stets and Reid, 2011). Clearly, more research is needed to disentangle changes in attention to stimuli effects from changes in underlying mechanism.

Nevertheless, the Nc shows face-house discrimination at both visits, coupled with a general increase in amplitude for both categories with age. Similar increases in amplitude have also been reported in one longitudinal study that tested the amplitude maturation of the Nc component in 4- to 12-month-olds (Webb et al., 2005). In contrast, a cross-sectional study that targeted 4.5-, 6- and 7.5-month-old infants (Guy et al., 2016) does not report such amplitude increases. It is possible that observing such amplitude increases requires a large interval. Note that by using a five-month-interval we contrast infants from early to late infancy, when face-processing undergoes dramatic changes (e.g. perceptual narrowing, Pascalis et al., 2002). Another possibility is that such increases only become apparent in larger longitudinal samples as in ours, because infants might vary substantially in their neuro-development (Johnson, 2001). In any case, our study with 80 infants at both five and 10 months reveals a general increase in cortical activity related to visual processing.

To summarize, while we did not observe any development specific to face-categorization, the peak-to-trough analyses reveal instead that at all components there was a comparable increase in infants’ cortical activity for both face and non-face stimuli from five to 10 months of age. It is possible that our choice of average reference affects amplitude of face-sensitive components (Joyce and Rossion, 2005), and hence could be a confounding factor. One limitation of the current research is that we could not use a different reference as we did not record mastoids. Nevertheless, our developmental effects are in line with previous research (also often using an average reference, e.g. Conte et al., 2020) suggesting that amplitude increases throughout infancy, before it decreases again in childhood (de Haan, 2007). Such amplitude changes have been previously linked to changes in synaptic density (Courchesne, 1990; Vaughan and Kurtzberg, 1992). Indeed, the infant brain undergoes substantial functional and structural changes during the first year of life: between four and six months of age there is a burst of synapse formation in the visual cortex, and around eight and 12 months of age there is the emergence of white matter in frontal, parietal and occipital regions (Johnson, 2001). Therefore, the amplitude changes between five and 10 months in our study might simply indicate a general increase in synaptic density and brain activity towards visual stimuli, which continues into adolescence (Kuefner et al., 2010).

### 4.2 Range in developmental trajectories across components

While all components testify to face-categorization already present at five months, the Markov models add information on its development by visualizing the variation in individual trajectories per component. We only compare Markov models for the P1, N290 and the Nc, since the Markov model for the P400 is very similar to that of the N290, which is unsurprising since peak-to-trough analyses reveal that our P400 mainly reflects carry-over effects from the N290. If a component marks development in face-categorization, one would expect it to be gradual. That is, we expect immature responses slowly to become more mature-like: i.e. with more infants moving into the dominant group of face-categorization at the second visit, while fewer infants moving out of the dominant group. We observe such a pattern for the N290, but not for the P1 and the Nc. Specifically, at the first visit the dominant N290 response comprises more cases (83 %) compared to the dominant P1 and Nc group responses (55 % and 60 %, respectively). Next, at the second visit there are more infants who remain in the dominant group for the N290 (88 % respectively) than for the P1 or the Nc (61 % and 67 %, respectively). Finally, there are fewer types of transitions between groups for the N290 than for the P1 and Nc. Notably, the dominant trajectory for the non-dominant groups (that is, those five-month-olds showing either no difference or a positive difference) is towards the dominant group for the N290 (93 %), whereas this trend is least apparent for the Nc (53 %). This suggests that the N290 might be well-developed, and hence less prone to individual variation, than the other components.

We speculate that the difference in variation across the individual trajectories among the components might relate to the nature of their underlying mechanisms. The N290 is an ERP component often associated with the encoding of faces (de Haan et al., 2003; Nelson and McCleery, 2008). It is possible that the early and extensive exposure to faces that infants’ experience swiftly contributes to the early emergence of this visual component and to its stability over time. As ERPs mainly reflect activation from the cortex (Luck, 2014), this reasoning is in line with a current developmental view on face processing. Johnson et al. (2015) suggests that cortical specialization emerges as the result of infants’ increased experience with faces, while it is also influenced by intrinsic biases to orient to faces and mechanisms of inter-regional connectivity.

It is noteworthy that there is more variation in the components not considered to be face-specific: the P1 and the Nc. As the P1 indexes low-level perceptual processing, atypical responses in the infant or child P1 has been linked to a variety of neurodevelopmental disorders (Hileman et al., 2011; Jones et al., 2018; Tye et al., 2013). In contrast, the Nc indexes attention allocation, preference or recognition (de Haan et al., 2003; Guy et al., 2016; Nelson and McCleery, 2008; Reynolds and Richards, 2005). All these processes associated with the Nc reflect higher-level processes of attention, which possibly compete with each other over the course of the experiment and across development. In other words, the Nc might not only pick up on the general face-house contrast, but might also fluctuate as it is sensitive to familiarity-novelty at the item-level (Stets and Reid, 2011). Consequently, the underlying mechanisms of the Nc may be less steady or there might be changes in attention allocation over time and across individuals compared to the visual processing indexed by N290. Indeed, variation in Nc responses has been linked to atypical development. For instance, toddlers with ASD show deviant Nc responses to faces (Dawson et al., 2012; Jones et al., 2016, 2017).

## 5 Conclusion

In summary, our findings indicate that compared to the Nc and P1, the N290 shows less variation in the trajectories in face-categorization, from five to 10 months of age. This finding suggests a difference in the underlying mechanisms. Source analysis studies also point to such difference, as these components originate in different cortical regions: whereas the N290 is localized in the middle fusiform gyrus, the infant P1 and Nc are localized in the lingual gyrus and parahippocampal gyrus respectively (e.g., Conte et al., 2020; Guy et al., 2016). A second finding is that our components of interest do not show any change in face-categorization in 5- to 10-month-olds, which indicates that face-categorization remains similar between these ages.

This work is meant as the first assessment of a larger dataset which aims to investigate how individual differences in face-categorization are linked to differences in the development of social cognition or social competence. Given that the N290 consistently appears to signal face-categorization longitudinally, we suggest that it is worthwhile to track development of those children that did not follow the dominant group patterns for the N290 in infancy. Nevertheless, studies aiming to grasp individual differences usually require outcomes with a maximum of between-participant variability (Hedge et al., 2018). It is therefore also possible that it is individual variation in the P1 or Nc rather than in the N290 that proves meaningful in explaining subsequent development. It therefore remains to be seen whether we observe such meaningful individual differences in face-categorization in the domain of perceptual processing (i.e., N290: Jones et al., 2016, 2017; McCleery et al., 2009), or in the domains of low-level sensory processing (i.e., P1: Hileman et al., 2011; Jones et al., 2018; Tye et al., 2013) or higher-cognitive processing (i.e., Nc: Jones et al., 2016).

## Declaration of Competing Interest

The authors report no declarations of interest.

## Footnotes

1 For statistical purposes, the Nc amplitude is usually calculated from an additional set of fronto-central electrodes (e.g., also FC1/2, Cz, C1/2). Unfortunately, we could not include these electrodes to study the Nc as the relevant electrodes were close to the CMS/DRL electrodes, often resulting in flat-line electrodes, which were subsequently removed from analysis. Therefore, our channel selection encompasses some of the relevant electrodes also included in previous relevant infant studies such as Kuefner et al., 2010; Munsters et al., 2017; van den Boomen et al., 2017. Note however that the Supplementary materials contains figures illustrating the grand averaged ERPs for faces and houses at all electrodes (including FC1/2, Cz, and C1/2) for those 75 infants who contribute data at both visits.

## References

[bib0005] Brainard D., Vision S. (1997). The psychophysics toolbox. Spat. Vis..

[bib0010] Conte S., Richards J.E. (2019). The development of face sensitive cortical processing in early infancy. Paper Presented at the Society for Research in Child Development.

[bib0015] Conte S., Richards J.E., Guy M.W., Xie W., Roberts J.E. (2020). Face-sensitive brain responses in the first year of life. NeuroImage.

[bib0020] Courchesne E., Rohrbaugh J.W., Parasuraman R., Johnson R. (1990). Chronology of postnatal human brain development: event-related potential, positron emission tomography, myelinogenesis, and synaptogenesis studies. Event Related Brain Potentials: Basic Issues and Applications.

[bib0025] Courchesne E., Ganz L., Norcia A.M. (1981). Event-related brain potentials to human faces in infants. Child Dev..

[bib0030] Dawson G., Webb S.J., McPartland J. (2005). Understanding the nature of face processing impairment in autism: insights from behavioral and electrophysiological studies. Dev. Neuropsychol..

[bib0035] Dawson G., Jones E.J., Merkle K., Venema K., Lowy R., Faja S. (2012). Early behavioral intervention is associated with normalized brain activity in young children with autism. J. Am. Acad. Child Adolesc. Psychiatry.

[bib0040] de Haan M., de Haan M. (2007). Current and future directions in infant electrophysiology. Infant EEG and Event-Related Potentials.

[bib0045] de Haan M., Nelson C.A. (1997). Recognition of the mother’s face by six‐month‐old infants: a neurobehavioral study. Child Dev..

[bib0050] de Haan M., Nelson C.A. (1999). Brain activity differentiates face and object processing in 6-month-old infants. Dev. Psychol..

[bib0055] de Haan M., Pascalis O., Johnson M.H. (2002). Specialization of neural mechanisms underlying face recognition in human infants. J. Cogn. Neurosci..

[bib0060] de Haan M., Johnson M.H., Halit H. (2003). Development of face-sensitive event-related potentials during infancy: a review. Int. J. Psychophysiol..

[bib0065] DeBoer T., Scott L.S., Nelson C.A. (2007). Methods for acquiring and analyzing infant event-related potentials. Infant EEG Event-Related Potentials.

[bib0070] Dering B., Martin C.D., Thierry G. (2009). Is the N170 peak of visual event-related brain potentials car-selective?. Neuroreport.

[bib0075] Frank M.C., Bergelson E., Bergmann C., Cristia A., Floccia C., Gervain J. (2017). A collaborative approach to infant research: promoting reproducibility, best practices, and theory‐building. Infancy.

[bib0080] Gliga T., Dehaene-Lambertz G. (2005). Structural encoding of body and face in human infants and adults. J. Cogn. Neurosci..

[bib0085] Guy M.W., Zieber N., Richards J.E. (2016). The cortical development of specialized face processing in infancy. Child Dev..

[bib0090] Guy M.W., Richards J.E., Tonnsen B.L., Roberts J.E. (2018). Neural correlates of face processing in etiologically-distinct 12-month-old infants at high-risk of autism spectrum disorder. Dev. Cogn. Neurosci..

[bib0095] Halit H., de Haan M., Johnson M.H. (2003). Cortical specialisation for face processing: face-sensitive event-related potential components in 3-and 12-month-old infants. Neuroimage.

[bib0100] Halit H., Csibra G., Volein A., Johnson M.H. (2004). Face‐sensitive cortical processing in early infancy. J. Child Psychol. Psychiatry.

[bib0105] Hedge C., Powell G., Sumner P. (2018). The reliability paradox: why robust cognitive tasks do not produce reliable individual differences. Behav. Res. Methods.

[bib0110] Hileman C.M., Henderson H., Mundy P., Newell L., Jaime M. (2011). Developmental and individual differences on the P1 and N170 ERP components in children with and without autism. Dev. Neuropsychol..

[bib0115] Jeantet C., Caharel S., Schwan R., Lighezzolo-Alnot J., Laprevote V. (2018). Factors influencing spatial frequencies extraction in faces: a review. Neurosci. Biobehav. Rev..

[bib0120] Johnson M.H. (2001). Functional brain development in humans. Nat. Rev. Neurosci..

[bib0125] Johnson M.H., Senju A., Tomalski P. (2015). The two-process theory of face processing: modifications based on two decades of data from infants and adults. Neurosci. Biobehav. Rev..

[bib0130] Jones E.J., Venema K., Earl R., Lowy R., Barnes K., Estes A. (2016). Reduced engagement with social stimuli in 6-month-old infants with later autism spectrum disorder: a longitudinal prospective study of infants at high familial risk. J. Neurodev. Disord..

[bib0135] Jones E.J., Venema K., Earl R.K., Lowy R., Webb S.J. (2017). Infant social attention: an endophenotype of ASD‐related traits?. J. Child Psychol. Psychiatry.

[bib0140] Jones E.J., Dawson G., Webb S.J. (2018). Sensory hypersensitivity predicts enhanced attention capture by faces in the early development of ASD. Dev. Cogn. Neurosci..

[bib0145] Joyce C., Rossion B. (2005). The face-sensitive N170 and VPP components manifest the same brain processes: the effect of reference electrode site. Clin. Neurophysiol..

[bib0150] Kuefner D., De Heering A., Jacques C., Palmero-Soler E., Rossion B. (2010). Early visually evoked electrophysiological responses over the human brain (P1, N170) show stable patterns of face-sensitivity from 4 years to adulthood. Front. Hum. Neurosci..

[bib0155] Langner O., Dotsch R., Bijlstra G., Wigboldus D., Hawk S., van Knippenberg A. (2010). Presentation and validation of the Radboud Faces Database. Cogn. Emot..

[bib0160] Libertus K., Landa R.J., Haworth J.L. (2017). Development of attention to faces during the first 3 years: influences of stimulus type. Front. Psychol..

[bib0165] Luck S.J. (2014). An Introduction to the Event-Related Potential Technique.

[bib0170] Luck S.J., Gaspelin N. (2017). How to get statistically significant effects in any ERP experiment (and why you shouldn’t). Psychophysiology.

[bib0175] Luyster R.J., Powell C., Tager-Flusberg H., Nelson C.A. (2014). Neural measures of social attention across the first years of life: characterizing typical development and markers of autism risk. Dev. Cogn. Neurosci..

[bib0180] Macchi Cassia V., Kuefner D., Westerlund A., Nelson C.A. (2006). A behavioural and ERP investigation of 3-month-olds’ face preferences. Neuropsychologia.

[bib0185] McCleery J.P., Akshoomoff N., Dobkins K.R., Carver L.J. (2009). Atypical face versus object processing and hemispheric asymmetries in 10-month-old infants at risk for autism. Biol. Psychiatry.

[bib0190] Munsters N.M., van Ravenswaaij H., van den Boomen C., Kemner C. (2017). Test-retest reliability of infant event related potentials evoked by faces. Neuropsychologia.

[bib0195] Nelson C.A., McCleery J.P. (2008). Use of event-related potentials in the study of typical and atypical development. J. Am. Acad. Child Adolesc. Psychiatry.

[bib0200] Norcia A.M., Tyler C.W. (1985). Spatial frequency sweep VEP: visual acuity during the first year of life. Vision Res..

[bib0205] Parise E., Handl A., Striano T. (2010). Processing faces in dyadic and triadic contexts. Neuropsychologia.

[bib0210] Pascalis O., de Haan M., Nelson C.A. (2002). Is face processing species-specific during the first year of life?. Science.

[bib0215] Peykarjou S., Hoehl S. (2013). Three-month-olds’ brain responses to upright and inverted faces and cars. Dev. Neuropsychol..

[bib0220] Peykarjou S., Pauen S., Hoehl S. (2014). How do 9‐month‐old infants categorize human and ape faces? A rapid repetition ERP study. Psychophysiology.

[bib0225] Picton T.W., Taylor M.J. (2007). Electrophysiological evaluation of human brain development. Dev. Neuropsychol..

[bib0230] Reynolds G.D., Richards J.E. (2005). Familiarization, attention, and recognition memory in infancy: an event-related potential and cortical source localization study. Dev. Psychol..

[bib0235] Rossion B., Jacques C. (2008). Does physical interstimulus variance account for early electrophysiological face sensitive responses in the human brain? Ten lessons on the N170. Neuroimage.

[bib0240] Rossion B., Gauthier I., Tarr M.J., Despland P., Bruyer R., Linotte S., Crommelinck M. (2000). The N170 occipito-temporal component is delayed and enhanced to inverted faces but not to inverted objects: an electrophysiological account of face-specific processes in the human brain. Neuroreport.

[bib0245] Scott L.S., Nelson C.A. (2006). Featural and configural face processing in adults and infants: a behavioral and electrophysiological investigation. Perception.

[bib0250] Stets M., Reid V.M. (2011). Infant ERP amplitudes change over the course of an experimental session: implications for cognitive processes and methodology. Brain Dev..

[bib0255] Taylor M.J., Batty M., Itier R.J. (2004). The faces of development: a review of early face processing over childhood. J. Cogn. Neurosci..

[bib0260] Tye C., Mercure E., Ashwood K.L., Azadi B., Asherson P., Johnson M.H. (2013). Neurophysiological responses to faces and gaze direction differentiate children with ASD, ADHD and ASD+ ADHD. Dev. Cogn. Neurosci..

[bib0265] van den Boomen C., Munsters N.M., Kemner C. (2017). Emotion processing in the infant brain: the importance of local information. Neuropsychologia.

[bib0270] van Onland-Moret, N. C., Buizer-Voskamp, J. E., Albers, M. E.W. A., Brouwer, R. M., Buimer, E. E.L., Hessels, R.S., de Heus, R., Huijding, J., Junge, C. M. M., Mandl, René C.W., Pas, P., Vink, M., van der Wal, J. J.M., Hulshoff Pol, H. E., & Kemner, C. Manuscript submitted for publication.

[bib0275] Vaughan H.G.Jr., Kurtzberg D., Gunnar M.R., Nelson C.A. (1992). Electrophysiological indices of human brain maturation and cognitive development.

[bib0280] Webb S.J., Long J.D., Nelson C.A. (2005). A longitudinal investigation of visual event‐related potentials in the first year of life. Dev. Sci..

[bib0285] Webb S.J., Jones E.J., Merkle K., Venema K., Greenson J., Murias M., Dawson G. (2011). Developmental change in the ERP responses to familiar faces in toddlers with autism spectrum disorders versus typical development. Child Dev..

[bib0290] Xie W., Richards J.E. (2016). Effects of interstimulus intervals on behavioral, heart rate, and event‐related potential indices of infant engagement and sustained attention. Psychophysiology.

[bib0295] Yrttiaho S., Forssman L., Kaatiala J., Leppänen J.M. (2014). Developmental precursors of social brain networks: the emergence of attentional and cortical sensitivity to facial expressions in 5 to 7 months old infants. PLoS One.

