# Supplementary material for: Charting development of ERP components on face-categorization: Results from a large longitudinal sample of infants
Source: Dev Cogn Neurosci. 2020 Aug 16;45:100840. doi: 10.1016/j.dcn.2020.100840 (PMC7476229; doi:10.1016/j.dcn.2020.100840)
Supplement: Supplementary file 1 [file mmc1.docx]

**Supplementary materials**

**A. Analyses on possible confounds of change between visits (or development)**

*Test of equal variances* – Separately for each component of interest, we tested whether the variances of the face-minus-house amplitude difference were similar between the two visits. The Brown-Forsythe tests shows that there is a similar variation (and therefore noise) between the face-house difference obtained at visit1 and visit2 for each component: P1: *F* (1,79) = .186, *p* = .67, *η_p_*^2^ = .002; N290: *F* (1,79) = 0.49, *p* = .49, *η_p_*^2^ = .006; P400: *F* (1,79) = 0.012, *p* = .91, *η_p_*^2^ = .0001; Nc: *F* (1,79) =0 .39, *p* = .53, *η_p_*^2^ = .005.

*Age difference at visit 1 and time interval between the two visits –*We measured age at the first visit (Age visit1) and the time-interval between the two visits (Age Difference) in days (see Table 1S). We performed the repeated measures ANOVAs reported in the main text [within subject factors: Visit (1, 2), Stimulus type (Face, House)] with Age visit1 and Age Difference as covariates. Only for the N290 component, there is a significant interaction between Age visit1 and Stimulus type, *F*(1,77) = 4.89, *p* = .030, *η_p_*^2^ = .060; there are no other significant interactions with the covariates for P1 (all *p*s>.13); N290 (all *p*s > .098), P400 (all *p*s > .095), and Nc (all *p*s > .27). We followed-up the Age visit1 by Stimulus type interaction found for N290 amplitudes by computing a Pearson’s correlation between Age visit1 and the mean of the face-house amplitude difference obtained at visit 1 and 2 (i.e., average of the two difference scores); this analyses revealed a weak negative correlation, *r* (80) = -.28, *p* = .013, which suggests a relation between age at first visit and general face-house difference. Yet, follow-up tests show no significant correlation either between Age visit1 and the face-house difference at visit 1 (*r* (80) = -.18, *p* = .12) or between Age visit1 and the face-house difference at visit 2 (*r* (80) = -.19, *p* = .085). The lack of significant correlations at either visit suggests nonetheless that there is no relation between the variance of age and the amplitude difference at one visit. Hence, for all components, there is no reason to believe that the lack of change in face sensitivity between the two visits emerged from the ANOVA is due to these two age factors.

|  | Age visit 1 | Age visit 2 | Age Difference between visits |
| --- | --- | --- | --- |
| P1/N290/P400 | 168 (22.5) | 319 (25.7) | 151 (34.2) |
| Nc | 168 (22.0) | 318 (25.7) | 150 (33.5) |

Table 1S. Mean age at visit 1 and 2, and mean age difference between the two visits of infants providing EEG data for the N290/P400 and for the Nc. Age is reported in days, standard deviations are shown in the brackets.

*Number of trials* – Table 2S reports the number of trials per condition, per visit, and per component. Separately for each component and for each visit, we computed Pearsons’ correlations between the face-house amplitude difference and the number of trials used to compute the house or the face mean amplitudes. Significant correlations are broken down to examine whether trial size is related to the processing of each category rather than the difference score. We do not adjust for Bonferroni corrections. If there is a relationship, we expect it to be present at both visits. Otherwise, if a relationship only holds at one visit but not at another visit, we interpret this as a Type-1 error.

P1–For both visits, there is no correlation between the face-house amplitude difference vs the number of house or face trials (all *p*s>.31).

N290–For visit 1, there is a negative correlation between the face-house amplitude difference vs. the number of house trials (*r*(80) = -.25, *p* = .022) as well as vs. the number of face trials (*r*(80) = -.28, *p* = .013). For visit 2, there is no significant relation between the face-house amplitude difference and the number of house or face trials, all *p*s > .28. When we break down this correlation for visit 1, we observe a weak positive correlation between the mean amplitude for house processing and number of trials of the house condition (*r*(80) = .27, *p* = .015), whereas there is no correlation between amplitude and number of trials of the face condition (*r*(80) = .015, *p* = .90). As follow-up tests show only a weak positive relation between the amplitude and the number of trials for the house condition at visit 1, while no effect emerged for the house condition at visit 2 nor for the face condition at any visits, we interpret this effect as spurious (Type I-error).

P400– For visit 1, we observe a negative correlation between the face-house amplitude difference vs. the number of house trials (*r*(80) = -.26, *p* = .020), as well as vs. the number of face trials (*r*(80) = -.31, *p* = .006). For visit 2, there is a weak negative correlation between the face-house amplitude difference and the number of face trials (*r*(80) = -.24, *p* = .032), while no correlation between the amplitude difference and the house trials (*r*(80) = -.071, *p* = .53). Follow-up correlations for visit 1 show a moderate positive correlation between the amplitude and number of trials of the house condition (*r* (80)= .41, *p* <.001), while there is no correlation between the amplitude and number of trials of the face condition (*r* (80)= .21, *p* = .059). Turning to visit 2, there is no correlation between the mean amplitudes and number of trials of either condition, all *p*s > .32. Similarly to N290, the analyses on P400 show a correlation between number of trials and amplitude of the house condition at visit1, which is not surprising since these two components are recorded from the same electrode sets. Since we do not find any other significant correlations we believe that it is unlikely that the number of trials acted as a confound to the ANOVA interaction.

Nc–There is no correlation between the face-house amplitude differences and the number of face or house trials for any visit (all *p*s > .20).

*All components–* We further computed the 2 (Visit) by 2 (Stimulus type) ANOVAs only including infants with a minimum of 20 trials per variable (n = 57) to test if a larger number of trials (as in the original ANOVAs) masks the Stimulus type by Visit interaction. Results are similar. First, for each component there is a main effect of Stimulus type (P1: *F*(1,56) = 31, *p* < .001, *η_p_*^2^ = .36; N290: *F*(1,56) = 170, *p* < .001, *η_p_*^2^ = .75; P400: *F*(1,56) = 146, *p* < .0001, *η_p_*^2^ = .72; Nc: *F*(1,55) = 36.5, *p* < .001, *η_p_*^2^ = .39). Second, we see a main effect of Visit for the N290, P400, and the Nc (N290: *F*(1,56) = 7.38, *p* = .009, *η_p_*^2^ = .12; P400: *F*(1,56) = 21.3, *p* < .0001, *η_p_*^2^= .27; Nc: *F*(1,55) = 10.5, *p* = .002, *η_p_*^2^= .16); this was not present for the P1 (*F*(1,56) = .046, *p* = 83, *η_p_*^2^ = .001). Third, there are no interactions between Stimulus type and Visit (all *p*s > .23).

Together these results suggest that the difference in number of house or face trials does not mask or influence the (lack of) change observed in the main ANOVAs investigating development of face categorization.

|  | N trials visit 1 | N trials visit 2 |
| --- | --- | --- |
| P1/N290/P400 Face | 31.2 (8.85) | 28.9 (8.73) |
| P1/N290/P400 House | 32.0 (8.32) | 28.8 (8.73) |
| Nc Face | 31.3 (8.75) | 28.7 (8.60) |
| Nc House | 32.1 (8.41) | 28.51 (8.64) |

Table 2S. Mean of trials averaged for each component in response to faces and houses, at visit 1 and 2. There is no statistical difference between the number of trials included for faces and houses at each time point (all ps > .096).

**B. Effect of Stimulus type, Visit, and Electrode on the amplitude of the P1, N290, P400 and Nc components.**

To further explore differences in face-categorization at electrode level, we repeated our main analyses including Electrode as within subject factor. Specifically, for the P1 we exported mean activity between 90-180ms after stimulus onset at the PO3, O1, Oz, O2, PO4 electrodes; for the N290 and P400 the mean amplitude between 170-300ms and 300-500ms respectively was exported from P3, PO3, O1, Oz, O2, PO4 electrodes; while for the Nc mean amplitude between 300-600ms was obtained from C3, C4, Fz. Using these electrodes, we performed a repeated measures ANOVA, with Electrode, Stimulus Type, Visit as independent variables, with mean activity as dependent variable. We also performed this ANOVA for the amplitude resulted from the peak-to-trough tests on P1, N290 and P400 components. Specifically, for each electrode, subject and visit, we subtracted from the components of interest the mean amplitude of the preceding peak; the obtained amplitude difference was used as dependent variable in the ANOVA. Note that for the peak-to-trough tests on P1, we subtracted the mean amplitude between 70-90ms post stimulus onset (i.e. N80) from the P1 amplitude. Main interest was in the effects of Stimulus type, and of Stimulus type and Visit per Electrode. Hence, if the three-way interaction or the Electrode by Stimulus type interaction were significant, we performed further ANOVAs and/or t-test investigating these effects. When appropriate we applied Wilcoxon Signed Rank Test instead of t-tests. All tests were corrected for multiple comparisons when applicable (i.e. alpha of .05 divided by number of comparisons).

Results are presented in Table 3S (P1), 4S (N290), 5S (P400; and P400-N290), and 6S (Nc).

*P1*– There was a three-way interaction. Further analyses show that houses elicited larger amplitudes than faces across both visits for the PO3, PO4 and O2; while this effect was only present at Visit2 for the O1 and Oz electrodes. Peak-to-trough tests confirm the three-way interaction, report the face-house difference at all electrodes across visits, and indicate larger amplitudes at second versus first visit for the occipital electrodes (i.e. O1, Oz, O2).

| P1 | | | | | |
| --- | --- | --- | --- | --- | --- |
| Comparison | *F* | | P value | *η_p_*^2^ |  |
| Electrode* Stimulus type* Visit | *F*(3.21,253.6) = 6.75 | | .0001* | .079 |  |
| Visit * Stimulus per Electrode | | | | | |
|  | Electrode | *F or t* (1,79) | P value | *η_p_*^2^ | Pairwise comparison |
|  | PO3 | 0.007 | .933 | <.001 |  |
|  | O1 | 5.91 | .017* | .07 |  |
|  | Oz | 4.38 | .04* | .053 |  |
|  | O2 | 3.81 | .055 | .046 |  |
|  | PO4 | 0.238 | .627 | .003 |  |
| Main effect of Stimulus | PO3 | 25.08 | <.001* | .241 | H > F |
|  | O1 | 15.26 | <.001* | .162 | H > F |
|  | Oz | 20.28 | <.001* | .204 | H > F |
|  | O2 | 17.55 | <.001* | .182 | H > F |
|  | PO4 | 25.62 | <.001* | .245 | H > F |
| Main effect of Visit | PO3 | 0.195 | .66 | .002 |  |
|  | O1 | 1.788 | .185 | .022 |  |
|  | Oz | 2.7 | .104 | .033 |  |
|  | O2 | 2.31 | .132 | .028 |  |
|  | PO4 | 0.715 | .400 | .009 |  |
| If interaction paired comparisons | O1 Visit1 | 0.591 | .556 |  |  |
| F vs H split by Visit | O1 Visit2 | 4.26 | <.001* |  | H > F |
|  | Oz Visit1 | 1.33 | .189 |  |  |
|  | Oz Visit2 | 4.13 | <.001* |  | H > F |
| P1-N80 Peak-to-trough tests | | | | | |
| Comparison | *F* | P value | *η_p_*^2^ |  |  |
| Electrode* Stimulus type* Visit | *F*(3.1,243) = 31.1 | .002* | .061 |  |  |
| Electrode* Stimulus type | *F*(2.95,233)=49.7 | .001* | .070 |  |  |
| Visit * Stimulus per Electrode | | | | | |
|  | Electrode | *F or t* (1,79) | P value | *η_p_*^2^ | Pairwise comparison |
|  | PO3 | 0.103 | .749 | .001 |  |
|  | O1 | 2.70 | .104 | .033 |  |
|  | Oz | 4.71 | .033* | .056 |  |
|  | O2 | 4.02 | .048* | .048 |  |
|  | PO4 | 0.138 | .711 | .002 |  |
| Main effect of Stimulus | PO3 | 28.51 | <.001* | .256 | H > F |
|  | O1 | 32.66 | <.001* | .293 | H > F |
|  | Oz | 39.02 | <.001* | .331 | H > F |
|  | O2 | 54.16 | <.001* | .407 | H > F |
|  | PO4 | 79.5 | <.001* | .502 | H > F |
| Main effect of Visit | PO3 | 1.45 | .232 | .018 |  |
|  | O1 | 6.58 | .012* | .077 | V1 < V2 |
|  | Oz | 9.52 | .003* | .108 | V1 < V2 |
|  | O2 | 7.50 | .008* | .087 | V1 < V2 |
|  | PO4 | .896 | .347 | .011 |  |
| If interaction |  | *t(1,79)* | P value |  |  |
| paired comparisons | Oz | 2.26 | .0123* |  | H > F |
| F vs H split by Visit | Oz | 5.71 | <.001* |  | H > F |
| alpha .0125 (4 comparisons) | O2 | 3.46 | .001* |  | H > F |
|  | O2 | 7.00 | <.001* |  | H > F |

Table 3S. Results of analyses on P1 and on amplitude resulting from P1-N80 peak-to-trough tests. Asterisks indicate significant effect.

*N290*– There was an interaction between Electrode, Stimulus Type and Visit. Planned comparisons report that faces elicited a more negative amplitude than Houses at both visits for all electrodes. In addition, at the PO3, O1, Oz, O2, and P4 electrodes there were higher amplitudes at Visit2 than at Visit1. Peak-to-trough analyses do not reveal the three-way interaction emerged from the previous test. Yet, follow-up tests on single electrodes confirm face-house differences across visits (i.e. faces eliciting more negative amplitudes than houses) at all sites and indicate higher amplitudes (i.e. more positive) to both stimuli at second compared to first visit for the PO3, O1, Oz, O2, PO4 electrodes.

| N290 | | | | | |
| --- | --- | --- | --- | --- | --- |
| Comparison | *F* | | P value | *η_p_*^2^ |  |
| Electrode* Stimulus type* Visit | *F*(3.69,291) = 3.62 | | .008* | .044 |  |
| Visit * Stimulus per Electrode | | | | | |
|  | Electrode | *F or t* (1,79) | P value | *η_p_*^2^ | Pairwise comparison |
|  | P3 | 1.73 | .193 | .021 |  |
|  | PO3 | 1.26 | .260 | .016 |  |
|  | O1 | 7.08 | .009* | .082 |  |
|  | Oz | 1.44 | .234 | .018 |  |
|  | O2 | 1.88 | .174 | .023 |  |
|  | PO4 | 1.39 | .241 | .017 |  |
|  | P4 | 0.088 | .770 | .001 |  |
| Main effect of Stimulus | P3 | 11.03 | .001* | .123 | H > F |
|  | PO3 | 110 | <.001* | .580 | H > F |
|  | O1 | 165 | <.001* | .676 | H > F |
|  | Oz | 164 | <.001* | .675 | H > F |
|  | O2 | 160 | <.001* | .291 | H > F |
|  | PO4 | 38.7 | <.001* | .329 | H > F |
|  | P4 | 121 | <.001* | .060 | H>F |
| Main effect of Visit | P3 | 2.5 | .118 | .031 |  |
|  | PO3 | 4.4 | .039* | .053 | V1 < V2 |
|  | O1 | 25.65 | <.001* | .245 | V1 < V2 |
|  | Oz | 35.1 | <.001* | .308 | V1 < V2 |
|  | O2 | 32.45 | <.001* | .291 | V1 < V2 |
|  | PO4 | 2.57 | .113 | .032 |  |
|  | P4 | 5.36 | .023* | .064 | V1 < V2 |
| If interaction paired comparisons | O1 Visit1 | 9.1 | <.001* |  | H > F |
| F vs H split by Visit | O1 Visit2 | 9.3 | <.001* |  | H > F |
| N290-P1 Peak-to-trough tests | | | | | |
| Comparison | *F* | | P value | *η_p_*^2^ |  |
| Electrode* Stimulus type* Visit | *F*(2.97,235) = 25.00 | | .154 | .020 |  |
| Electrode* Visit | *F*(2.45,194) = 27.92 | | <.001* | .261 |  |
| Electrode* Stimulus type | *F*(2.78,221) = 59.08 | | <.001* | .428 |  |
| Visit * Stimulus per Electrode | | | | | |
|  | Electrode | *F or t* (1,79) | P value | *η_p_*^2^ | Pairwise comparison |
|  | P3 | 3.87 | .053 | .047 |  |
|  | PO3 | 2.65 | .107 | .032 |  |
|  | O1 | 1.164 | .284 | .015 |  |
|  | Oz | 12.44 | .267 | .607 |  |
|  | O2 | 0.028 | .868 | .0003 |  |
|  | PO4 | 0.028 | .868 | .0003 |  |
|  | P4 | 0.191 | .664 | .002 |  |
| Main effect of Stimulus | P3 | 8.51 | .005* | .097 | H > F |
|  | PO3 | 94.02 | <.001* | .543 | H > F |
|  | O1 | 142 | <.001* | .642 | H > F |
|  | Oz | 126 | <.001* | .615 | H > F |
|  | O2 | 140 | <.001* | .639 | H > F |
|  | PO4 | 77.09 | <.001* | .494 | H > F |
|  | P4 | 19.36 | <.001* | .197 | H > F |
| Main effect of Visit | P3 | .081 | .78 | .001 |  |
|  | PO3 | 18.98 | <.001* | .194 | V1 < V2 |
|  | O1 | 34.93 | <.001* | .307 | V1 < V2 |
|  | Oz | 56.62 | <.001* | .418 | V1 < V2 |
|  | O2 | 45.54 | <.001* | .366 | V1 < V2 |
|  | PO4 | 28.12 | <.001* | .263 | V1 < V2 |
|  | P4 | 0.080 | .778 | .001 |  |

Table 4S. Results of analyses on N290 and on amplitude resulting from N290-P1 peak-to-trough tests. Asterisks indicate significant effect.

*P400*– There was a marginal three-way interaction (*p*=.057) and significant Electrode by Stimulus type interaction (*p*<.001). Further tests indicate amplitude differences between house and faces across visits for most electrodes (i.e. PO3, O1, Oz, O2, PO4, P4), while only for P3 this effect emerged at Visit2 but not at Visit1. In addition, larger amplitudes were obtained at Visit2 compared to Visit1 for the PO and O electrodes. Peak-to-trough analyses confirmed the three-way interaction emerged from the P400 tests. Differences between the amplitudes evoked by faces and houses are found again for the PO3 and PO4 electrodes across both visits, while for theP3, Oz, and P4 electrodes this difference was significant only at Visit2; no difference reached significant for electrodes O1 and O2. The increased amplitude observed at the P400 from Visit1 to Visit 2 was confirmed also by these tests for most electrodes (see table 5S).

| P400 | | | | | |
| --- | --- | --- | --- | --- | --- |
| Comparison | *F* | | P value | *η_p_*^2^ |  |
| Electrode* Stimulus type* Visit | *F*(4.16,326) = 2.29 | | .057 | .028 |  |
| Electrode* Stimulus type | *F*(4.32,341)= 21.8 | | <.001* | .217 |  |
| Visit * Stimulus per Electrode | | | | | |
|  | Electrode | *F or t* (1,79) | P value | η_2_ | Pairwise comparison |
|  | P3 | 7.79 | .007* | .090 |  |
|  | PO3 | 0.005 | .944 | .001 |  |
|  | O1 | 0.182 | .627 | .002 |  |
|  | Oz | 0.4 | .529 | .005 |  |
|  | O2 | 0.129 | .721 | .002 |  |
|  | PO4 | 0.189 | .665 | .002 |  |
|  | P4 | 0.076 | .784 | .001 |  |
| Main effect of Stimulus | P3 | 25.96 | <.001* | .248 | H > F |
|  | PO3 | 131 | <.001* | .624 | H > F |
|  | O1 | 163 | <.001* | .674 | H > F |
|  | Oz | 142 | <.001* | .644 | H > F |
|  | O2 | 128 | <.001* | .618 | H > F |
|  | PO4 | 113 | <.001* | .589 | H > F |
|  | P4 | 61.13 | <.001* | .436 | H > F |
| Main effect of Visit | P3 | 2.45 | .122 | .03 |  |
|  | PO3 | 23.44 | <.001* | .229 | V1 < V2 |
|  | O1 | 34.7 | <.001* | .305 | V1 < V2 |
|  | Oz | 33.13 | <.001* | .295 | V1 < V2 |
|  | O2 | 39.29 | <.001* | .332 | V1 < V2 |
|  | PO4 | 14.31 | <.001* | .153 | V1 < V2 |
|  | P4 | 0.054 | 817 | .001 |  |
| If two-way interaction |  | r*s* | P value |  |  |
| paired comparisons | P3 Visit1 | -1.76 | .078 |  |  |
| F vs H split by Visit | P3 Visit2 | -6.59 | <.001* |  | H > F |
| P400-N290 Peak-to-trough tests | | | | | |
| Comparison | *F* | | P value | *η_p_*^2^ |  |
| Electrode* Stimulus type* Visit | *F*(4.16,326) = 2.29 | | <.001* | .098 |  |
| Electrode* Stimulus type | *F*(3.69,292)=9.38 | | <.001* | .106 |  |
| Visit * Stimulus per Electrode | | | | | |
|  | Electrode | *F or t* (1,79) | P value | *η_p_*^2^ | Pairwise comparison |
|  | P3 | 10.88 | .001* | .121 |  |
|  | PO3 | 2.78 | .099 | .034 |  |
|  | O1 | 8.51 | .005* | .097 |  |
|  | Oz | 6.18 | .015* | .073 |  |
|  | O2 | 2.06 | .154 | .026 |  |
|  | PO4 | 0.051 | .821 | .001 |  |
|  | P4 | 5.21 | .025* | .062 |  |
| Main effect of Stimulus | P3 | 9.46 | .003* | .107 | H > F |
|  | PO3 | 6.39 | .013* | .075 | H > F |
|  | O1 | 0.205 | .652 | .003 |  |
|  | Oz | 6.79 | .011* | .079 | H < F |
|  | O2 | 1.26 | .265 | .016 |  |
|  | PO4 | 4.22 | .043* | .051 | H > F |
|  | P4 | 20.04 | <.001* | .202 | H > F |
| Main effect of Visit | P3 | 16.22 | <.001* | .170 | V1 < V2 |
|  | PO3 | 26.94 | <.001* | .254 | V1 < V2 |
|  | O1 | 6.14 | .015* | .072 | V1 < V2 |
|  | Oz | 1.43 | .235 | .018 |  |
|  | O2 | 2.09 | .152 | .026 |  |
|  | PO4 | 10.95 | .001* | .122 | V1 < V2 |
|  | P4 | 5.36 | .023* | .064 | V1 < V2 |
| If interaction |  | r*s* | P value |  |  |
| paired comparisons | P3 Visit1 | 1.09 | .625 |  |  |
| F vs H split by Visit | P3 Visit2 | -4.1 | <.001* |  | H > F |
| alpha .0125 (4 comparisons) | O1 Visit1 | -1.97 | .048 |  |  |
|  | O1 Visit2 | 2.35 | .019 |  |  |
|  | Oz Visit1 | -0.678 | .498 |  |  |
|  | Oz Visit2 | 3.377 | .001* |  | H < F |
|  | P4 Visit1 | -1.85 | .063 |  |  |
|  | P4 Visit2 | -4.31 | <.001* |  | H > F |

Table 5S. Results of analyses on P400 and on amplitude resulting from P400-N290 peak-to-trough tests. Asterisks indicate significant effect.

*Nc*–There was a significant Electrode by Stimulus type interaction (*p*<.001). Further tests indicate face-house differences at Central electrodes only.

| Nc | | | | | |
| --- | --- | --- | --- | --- | --- |
| Comparison | *F* | | P value | *η_p_*^2^ |  |
| Electrode* Stimulus type* Visit | *F*(1.78,141) = 2.87 | | 0.066 | .035 |  |
| Electrode* Stimulus type | *F(1.91,151)=19.74* | | < .001* | 0.2 |  |
| Stimulus (average across visits) per Electrode | | | | | |
|  | Electrode | *t* (1,79) | P value |  | Pairwise comparison |
| Main effect of Stimulus | C3 | -2.73 | 0.008* |  | F > H |
|  | Fz | -7.74 | 0.399 |  |  |
|  | C4 | -0.819 | <.001* |  | F > H |

Table 6S. Results of analyses on Nc. Asterisks indicate significant effect.

**C. Analyses on Peak latency of P1 (face and house); and of N290 (face).**

Here we repeat the analyses reported in the main text on the P1 latency to faces and houses, and N290 latency to faces, yet including Electrode sites as factor. Recall that peak latency was not reliably detected for the house condition of the N290, as well as for the house and face conditions of the P400 and Nc components, as these were rather broad waveforms instead of clear peaks. Therefore, we do not include tests on the latencies of these variables.

*P1*–Peak latency was extracted between 90-180ms after stimulus onset at the PO3, O1, Oz, O2, PO4 electrodes, separately for each condition and visit. We performed repeated measures ANOVA with latency as dependent variable and Stimulus Type, Visit and Electrode as within subject factor to further tests possible differences in face-house latency at each electrode site. If the three-way interaction was significant, we performed further ANOVAs and t-tests investigating face-house differences within electrodes; non-parametric testing (i.e. Wilcoxon Signed Rank Test) has been applied to paired comparisons when variables were not normally distributed. All tests were corrected for multiple comparisons when appropriate (i.e. .05 divided by number of comparisons).

Results of the ANOVA including Electrode as factor and relative follow-up tests are reported in table 7S: there was a significant three-way interaction (*F*(3.36,265) = 2.67; *p*=.042; η_p_^2^=.033); follow-up tests indicate a shorter latency for houses compared to faces only at Oz at the first Visit (*Median*_House_=114; *Median*_House_=129), this difference was not present at Visit2 (*p*>.05).

| P1 Latency | | | | | |
| --- | --- | --- | --- | --- | --- |
| Comparison | *F* | | P value | η_p_^2^ |  |
| Electrode* Stimulus type* Visit | *F*(3.36,265) = 2.67 | | .042* | .033 |  |
| Visit * Stimulus per Electrode | | | | | |
|  | Electrode | *F or t* (1,79) | P value | η_p_^2^ | Pairwise comparison |
|  | PO3 | 2.82 | .097 | .029 |  |
|  | O1 | 0.149 | .701 | .002 |  |
|  | Oz | 5.14 | .026* | .061 |  |
|  | O2 | 0.385 | .537 | .005 |  |
|  | PO4 | 0.001 | .977 | <.001 |  |
| Main effect of Stimulus | PO3 | 1.57 | .214 | .019 |  |
|  | O1 | 0.433 | .513 | .005 |  |
|  | Oz | 5.65 | .020* | .067 | H < F |
|  | O2 | 0.579 | .449 | .007 |  |
|  | PO4 | 0.13 | .719 | .002 |  |
| Main effect of Visit | PO3 | 2.32 | .131 | .029 |  |
|  | O1 | 0.605 | .439 | .008 |  |
|  | Oz | 0.02 | .888 | <.001 |  |
|  | O2 | 1.20 | .276 | .015 |  |
|  | PO4 | 5.76 | .019* | .068 | V2<V1 |
| If interaction paired comparisons |  | r*s* | P value |  |  |
| F vs H split by Visit | Oz Visit1 | -2.66 | .008* |  | H < F |
|  | Oz Visit2 | -1 | .317 |  |  |

Table 7S. Results of analyses on P1 latency at each Electrode sites. Asterisks indicate significant effects.

*N290*– Peak latency to face stimuli was extracted between 170-300ms after stimulus onset at the P3, PO3, O1, Oz, O2, PO4, P4 electrodes, separately for each visit and participant. We tested possible changes in peak latency to faces between visits for each electrode, by performing a repeated measures ANOVA with Electrode, and Visit as independent variables, and with peak latency as dependent variable. If the two-way interaction was significant, we performed paired comparisons between the latency obtained at each visit, separately for each electrode. We applied Wilcoxon Signed Rank Test when variables were not normally distributed. All tests were corrected for multiple comparisons when applicable (i.e. alpha of .05 divided by number of comparisons).

Results indicate a significant interaction between Visit and Electrode (*F*(6, 474) = 2.38; *p*=.046; η_p_^2^= .029). Follow-up tests report no significant effect of Visit at any Electrode (see Table 8S for all results). It is noteworthy that uncorrected results indicate an increase in peak latency from first to second visit at occipital electrodes only (O1, Oz, O2).

| N290 Latency (Face) | | | | |
| --- | --- | --- | --- | --- |
| Comparison | *F* | | P value | η_p_^2^ |
| Electrode* Visit | *F*(6, 474) = 2.38 | | .046* | 0.029 |
| Visit per Electrode | | | | |
|  | Electrode | r*s* | P value |  |
| Against alpha .007 | P3 | -0.62 | 0.536 |  |
| 7 comparisons | PO3 | -0.241 | 0.81 |  |
|  | O1 | 2.07 | 0.038 | V1<V2 |
|  | Oz | 1.96 | 0.049 | V1<V2 |
|  | O2 | 1.93 | 0.054 | V1<V2 |
|  | PO4 | -0.045 | 0.964 |  |
|  | P4 | -0.308 | 0.758 |  |

Table 8S. Results of analyses on N290 latency at each Electrode sites. Asterisks indicate significant effects.

**D. ERPs at all electrodes**

We plot for all 32 electrodes the grand averages of (the majority of) the 75 infants who contribute data for all components, separately for visit 1 (Figure 1S) and visit 2 (Figure 2S). Note that each electrode plots the grand averages of those infants who contributed 10 or more trials per stimulus type for the electrodes of interest– for the non-critical electrodes not all infant always contributed enough data points, which is why we omitted these electrodes from statistical analyses.


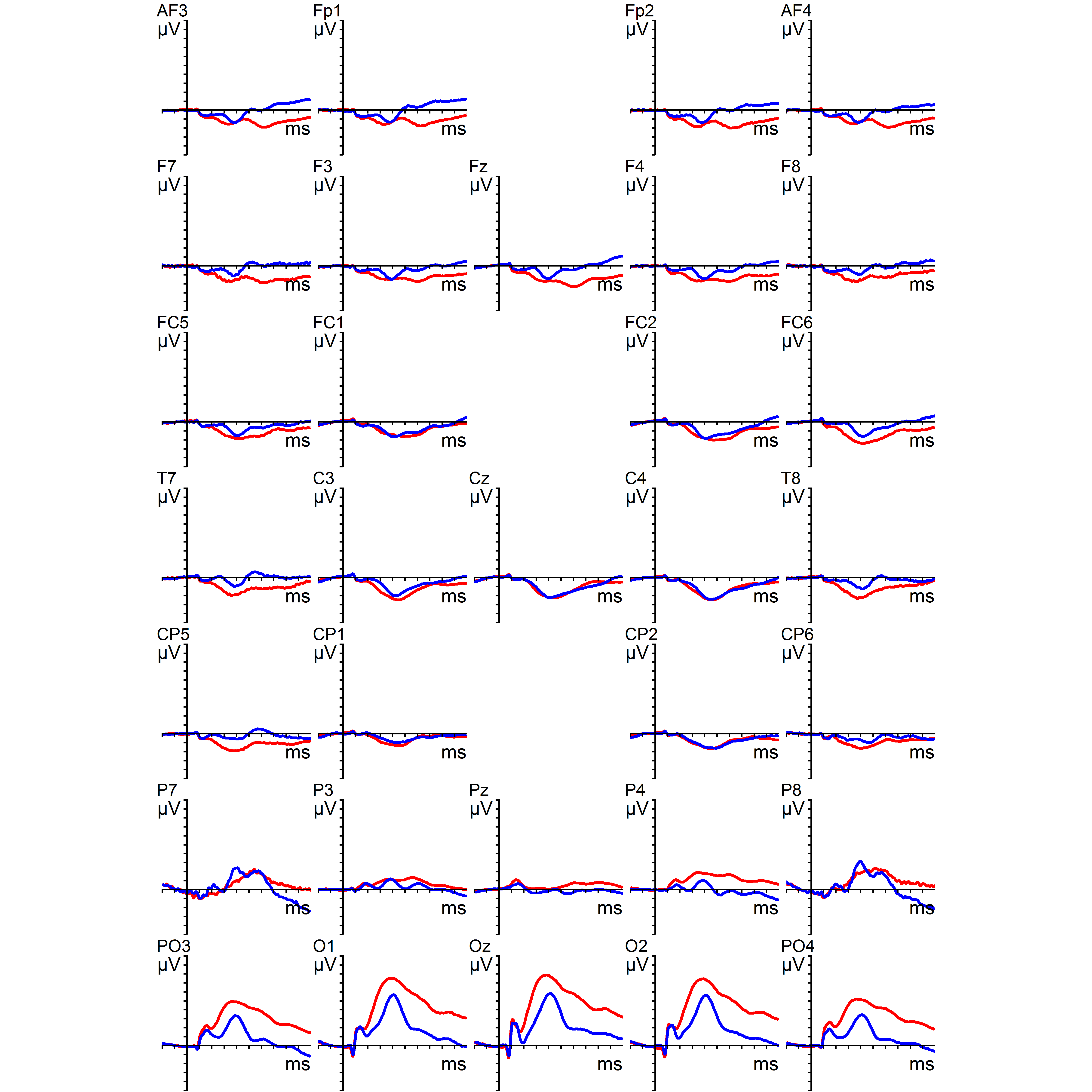


Figure 1S. Group averaged waveforms collected from 75 infants at first visit, in response to faces (blue line) and houses (red line). Tick marks on x-axis indicate 100ms, while on y-axis 100µV.


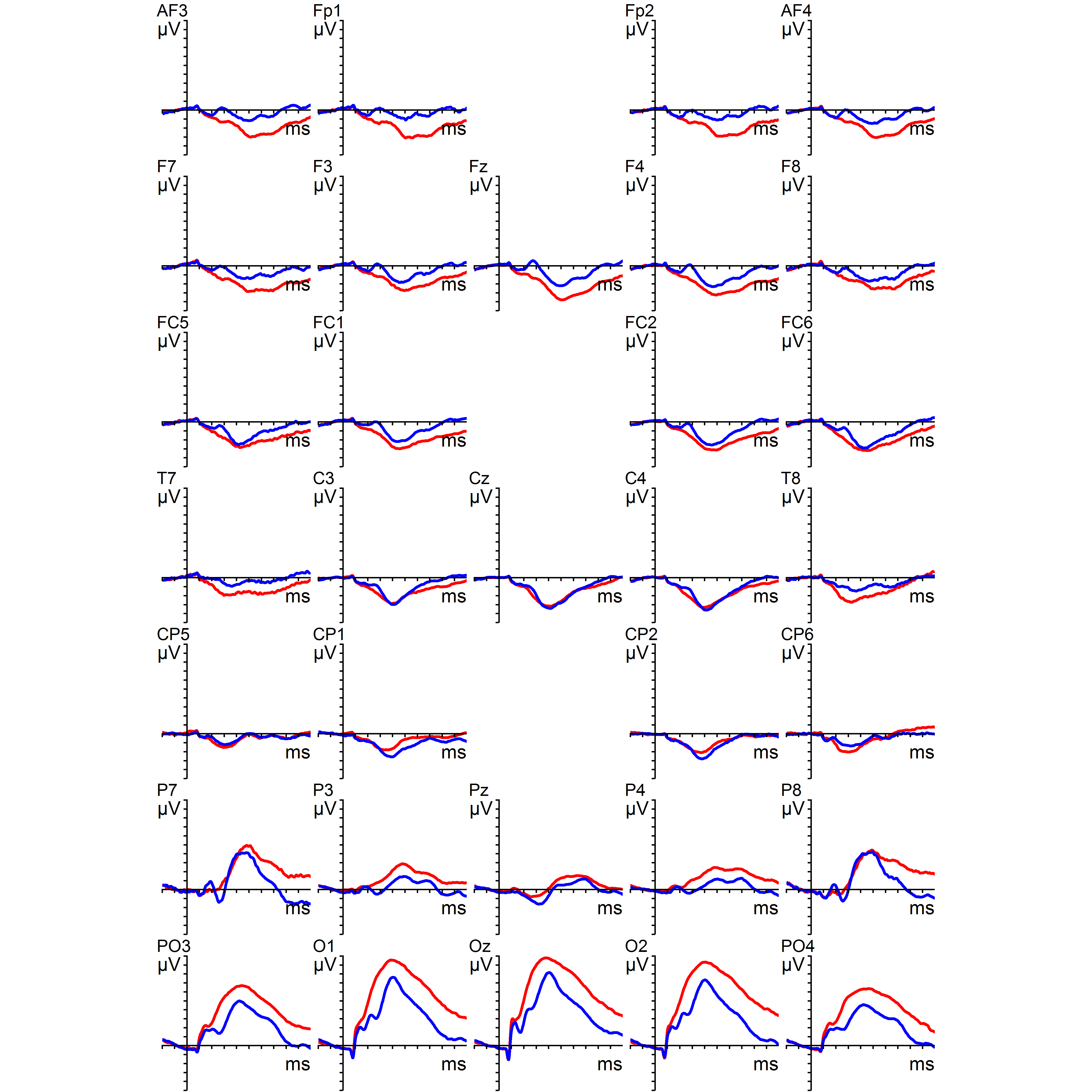


Figure 2S. Group averaged waveforms collected from 75 infants at second visit, in response to faces (blue line) and houses (red line). Tick marks on x-axis indicate 100ms, while on y-axis 100µV.

**E. Set of stimuli used as Houses and Faces.**

Here we report the full set of house and face stimuli used in the data collection in the current study. House pictures were taken from the internet, while face-stimuli were selected from the Radboud Faces Database (females identities: 12, 22, 26, 27, 37, 61; males identities: 7, 15, 25, 36, 49, 71; Langner et al., 2010).


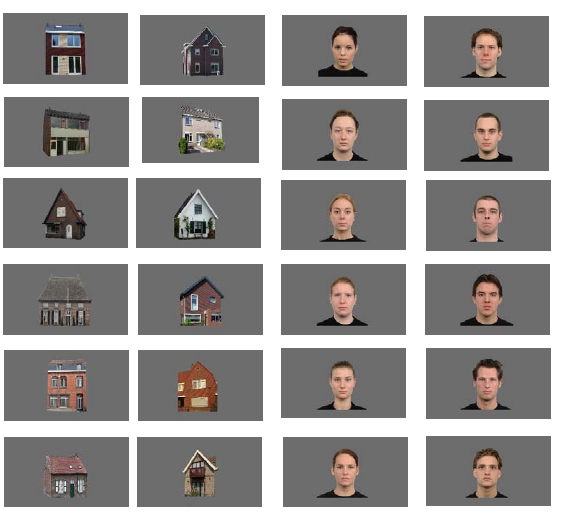


Figure 3S. Full set of stimuli used in the current study.
